# Single-base m^6^A epitranscriptomics reveals novel HIV-1 host interaction targets in primary CD4^+^ T cells

**DOI:** 10.1128/jvi.01536-25

**Published:** 2025-10-14

**Authors:** Siyu Huang, Yutao Zhao, Stacia Phillips, Julia E. Warrick, Michael G. Kearse, Chuan He, Li Wu

**Affiliations:** 1 Department of Microbiology and Immunology, Carver College of Medicine, The University of Iowa 311821, Iowa City, Iowa, USA; 2 Department of Chemistry, Institute for Biophysical Dynamics, University of Chicago 189300, Chicago, Illinois, USA; 3 Department of Biochemistry and Molecular Biology, Institute for Biophysical Dynamics, University of Chicago 311544, Chicago, Illinois, USA; 4 Department of Biological Chemistry and Pharmacology, Center for RNA Biology, The Ohio State University 167905 , Columbus, Ohio, USA; 5 Howard Hughes Medical Institute, University of Chicago 2462, Chicago, Illinois, USA; Icahn School of Medicine at Mount Sinai, New York, New York, USA

**Keywords:** HIV-1 infection, primary CD4^+^T cells, *N*^6^-methyladenosine (m^6^A), m^6^A-SAC-seq, perilipin 3 (PLIN3), polysome fractionation, translation

## Abstract

*N*^6^-methyladenosine (m^6^A) plays a critical role in regulating RNA Ostability, localization, and gene expression. m^6^A modification is also important for modulating the expression of viral and cellular genes during HIV-1 infection. However, the function of m^6^A modification in regulating HIV-1 infection of primary CD4^+^ T cells remains unclear. Here, we demonstrate that HIV-1 infection of activated primary CD4^+^ T cells promotes the interaction between the m^6^A writer complex subunits methyltransferase-like 3 and 14 (METTL3/METTL14). Using single-base m^6^A-specific RNA sequencing, we identified differentially m^6^A-modified cellular mRNAs in HIV-1-infected primary CD4^+^ T cells, including *perilipin 3* (*PLIN3*). We also identified 30 m^6^A sites in HIV-1 RNA from infected primary CD4^+^ T cells. HIV-1 infection increased *PLIN3* mRNA level and nuclear accumulation but decreased PLIN3 protein expression in primary CD4^+^ T cells. Polysome profiling revealed that *PLIN3* mRNA was less actively translated during HIV-1 infection of primary CD4^+^ T cells. Furthermore, PLIN3 knockdown in primary CD4^+^ T cells significantly reduced HIV-1 release but enhanced virion infectivity. Our results highlight the importance of m^6^A RNA modification during HIV-1 infection and suggest PLIN3 as a regulatory protein of HIV-1 replication in primary CD4^+^ T cells.

**IMPORTANCE:**

m^6^A is a common chemical modification on mRNA that regulates RNA stability, localization, and gene expression. m^6^A modification of viral and cellular RNA is important for HIV-1 infection. We found that HIV-1 infection of primary CD4^+^ T cells promotes the interaction between the m^6^A writer complex subunits that add m^6^A modification. Using m^6^A-specific RNA sequencing, we identified several cellular mRNAs with altered m^6^A modifications during HIV-1 infection, including *PLIN3*. Interestingly, HIV-1 infection increased *PLIN3* mRNA levels and nuclear localization but reduced PLIN3 protein expression in primary CD4^+^ T cells. When we knocked down PLIN3 in primary CD4^+^ T cells, it decreased HIV-1 release but made the HIV-1 more infectious. Our findings show the importance of m^6^A RNA modification in HIV-1 infection by regulating host genes like *PLIN3* and suggest a unique regulatory mechanism in HIV-1-infected primary CD4^+^ T cells.

## INTRODUCTION

*N*^6^-methyladenosine (m^6^A) is the most prevalent modification found in eukaryotic RNA, and it reversibly regulates gene expression by influencing RNA stability, alternative splicing, and protein translation (1, 2). This reversible modification is regulated by two groups of proteins involving a writer complex (methyltransferase) and erasers (demethylases). The m^6^A writer core complex consists of the catalytic subunit methyltransferase-like 3 (METTL3) and methyltransferase-like 14 (METTL14), which stabilizes METTL3 for substrate RNA binding (3–5). m^6^A erasers include fat mass and obesity-associated protein (FTO) and AlkB family member 5 (ALKBH5), which remove the methyl group (6, 7).

HIV-1 upregulates cellular m^6^A RNA levels in many target cell lines (8–12). In addition, we reported that cellular RNA m^6^A level is increased in peripheral blood mononuclear cells (PBMCs) from HIV-1-infected patients, and this effect is reversed by antiretroviral therapy (13). However, the mechanism of this cellular RNA m^6^A increase is not understood. Our previous studies showed no change in the expression levels of m^6^A writer and eraser proteins in primary CD4^+^ T cells from healthy donors (8) or latently HIV-1-infected J-Lat CD4^+^ T cells (12). Thus, we hypothesize that the increase in cellular m^6^A levels is regulated at the level of writer complex formation or methyltransferase activity.

Several studies have identified m^6^A modifications on both cellular mRNA and HIV-1 RNA in various cell lines using m^6^A RNA immunoprecipitation (meRIP) or crosslinking immunoprecipitation (CLIP) sequencing (reviewed in reference 14). However, these methods are not of sufficient resolution to identify the exact site of m^6^A. Identifying the precise location of and quantitative changes in m^6^A modifications in specific cellular transcripts during HIV-1 infection is important for understanding how m^6^A modifications modulate cellular RNAs and HIV-1 infection. The cellular m^6^A epitranscriptomic profile at single-base resolution has been reported for J-Lat CD4^+^ T cells under conditions of latency reversal (12). However, it is important to define the m^6^A landscape in primary CD4^+^ T cells, which are the major target of HIV-1 infection *in vivo*.

In this study, we examined how HIV-1 infection affects cellular mRNA m^6^A levels and characterized the m^6^A epitranscriptomic profile at single-base resolution in HIV-1-infected primary CD4^+^ T cells. We show that HIV-1 increased cellular mRNA m^6^A levels and promoted the interaction between METTL3 and METTL14 in CD4^+^ T cells. Additionally, we identified specific changes in m^6^A modifications in a subset of cellular transcripts in HIV-1-infected primary CD4^+^ T cells compared with mock controls. As an example, the mRNA encoding for perilipin 3 (PLIN3) has significantly higher levels of m^6^A modification at a single site in HIV-1-infected cells compared with mock-infected controls. We further demonstrate that HIV-1 infection regulates *PLIN3* mRNA and protein levels, and knocking down PLIN3 reduces HIV-1 release but enhances virion infectivity in primary CD4^+^ T cells. These phenotypes are cell-specific and not observed in Jurkat T cells. These findings suggest that differential m^6^A modification is a key regulatory mechanism in HIV-1 infection and provides the basis for future functional studies into how differential m^6^A modification affects HIV-1 replication.

## RESULTS

### HIV-1 upregulates m^6^A modification levels in cellular mRNA and promotes the interaction between METTL3 and METTL14 in CD4^+^ T cells

**Fig 1:**
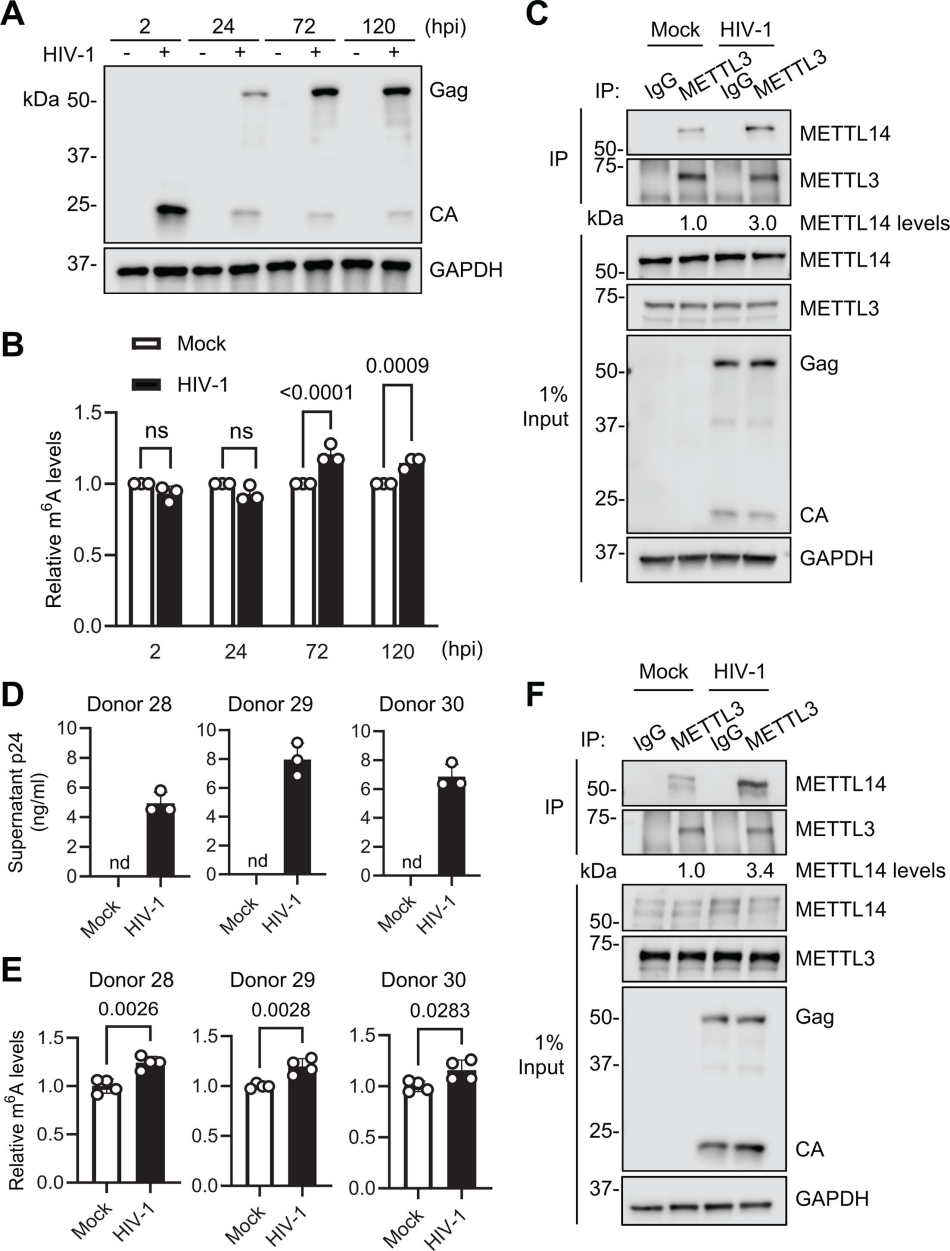
HIV-1 upregulates m^6^A modification levels in cellular mRNA and promotes the interaction between METTL3 and METTL14 in CD4^+^ T cells. (**A–C**) Jurkat cells were mock-infected or infected with X4-tropic HIV-1_NL4–3_ at an MOI of 1. (**A**) Infection was confirmed by immunoblot (IB) analysis of HIV-1 Gag and capsid (CA) at the indicated times post-infection. (**B**) m^6^A levels in cellular mRNA from mock or HIV-1-infected cells were measured by ELISA at the indicated times post-infection. The level of mock control was set as 1. (**C**) Immunoprecipitation (IP) was performed at 72 hpi using non-specific IgG or an anti-METTL3 antibody. The indicated proteins were detected by IB in the input and IP samples. Relative levels of METTL14 in the IP were determined by densitometry (METTL14/METTL3). (**D–F**) Activated primary CD4^+^ T cells were mock-infected or infected with HIV-1_NL4–3_ at an MOI of 1 for 96 h. (**D**) Infection was confirmed by measuring supernatant p24 levels by ELISA. nd, not detectable. (**E**) m^6^A levels in cellular mRNA from mock or HIV-1-infected cells were measured by ELISA. (**F**) IP was performed using non-specific IgG or an anti-METTL3 antibody. The indicated proteins were detected by IB in the input and IP samples. Relative levels of METTL14 in the IP were determined by densitometry (METTL14/METTL3). Data are shown as mean ± SD from three individual experiments. Two-way ANOVA with Bonferroni correction (**B**) and two-tailed, unpaired *t*-test (**E**) were used for statistical analysis (*P* values are shown on figures). ns, not significant.

We have reported that HIV-1 infection upregulates m^6^A levels of total cellular RNA in CD4^+^ T cells without changing the expression of m^6^A writers and erasers (8, 12). However, the mechanism of the m^6^A increase remains to be investigated. In the m^6^A writer complex, METTL3 is the catalytic subunit, whereas METTL14 binds to RNA substrate and stabilizes the m^6^A writer complex (4, 5). Therefore, we hypothesized that the METTL3/14 interaction may be increased during HIV-1 infection. We first determined the kinetics of m^6^A upregulation in response to X4-tropic HIV-1_NL4-3_ infection of Jurkat cells to identify the optimal time of infection at which to measure the METTL3/14 interaction. As expected, the expression of HIV-1 proteins increased over a period of 120 h (Fig. 1A). Measurement of m^6^A levels in polyadenylated RNA showed that there was a similar increase in infected cells at both 72 and 120 h post-infection (hpi) compared with mock-infected controls. (Fig. 1B). Next, we tested whether the interaction between METTL3 and 14 could be changed by HIV-1 infection in Jurkat cells. A METTL3 antibody was used for immunoprecipitation (IP), and IgG was used as a negative control. We observed a 3-fold increase in the amount of METTL14 that co-immunoprecipitated with METTL3 in HIV-1-infected Jurkat cells compared with mock-infected controls (Fig. 1C).

We then confirmed these results in primary CD4^+^ T cells isolated from the PBMC of three healthy blood donors. Productive infection of activated primary CD4^+^ T cells with X4-tropic HIV-1_NL4-3_ was confirmed using p24 enzyme-linked immunosorbent assay (ELISA) (Fig. 1D). m^6^A levels were measured, and we observed a small but significant increase for each donor (Fig. 1E). Total protein from these cells was used to perform co-immunoprecipitation (co-IP) as described above (Fig. 1F). We consistently observed a ~ 3-fold increase in the amount of METTL14 that co-immunoprecipitated with METTL3 in HIV-1-infected cells compared with mock-infected controls. We also observed significant increases in m^6^A levels in primary CD4^+^ T cells upon infection with an R5-tropic HIV-1_NLAD8_ strain (15) (Fig. S1). Overall, our results suggest that HIV-1 infection of CD4^+^ T cells causes an increase in the interaction between METTL3 and 14 that is associated with an increase in m^6^A levels.

### m^6^A-SAC-seq identifies m^6^A modifications in both cellular mRNAs and HIV-1 RNA

To identify the m^6^A sites in HIV-1-infected primary CD4^+^ T cells at single-base resolution, we used m^6^A-selective allyl chemical labeling and sequencing (m^6^A-SAC-seq) (16, 17). Primary CD4^+^ T cells isolated from PBMC from three individual healthy donors were infected with HIV-1 at a multiplicity of infection (MOI) of 1 for 96 h. Polyadenylated RNA was purified with two rounds of poly(A)-enrichment prior to m^6^A-SAC-seq. Sequencing data were analyzed to identify individual m^6^A sites that are significantly changed in HIV-1-infected cells compared with mock controls (Table S1). This analysis revealed 31,075 individual m^6^A modifications on 6,149 unique transcripts (Gene Expression Omnibus #280563).

**Fig 2:**
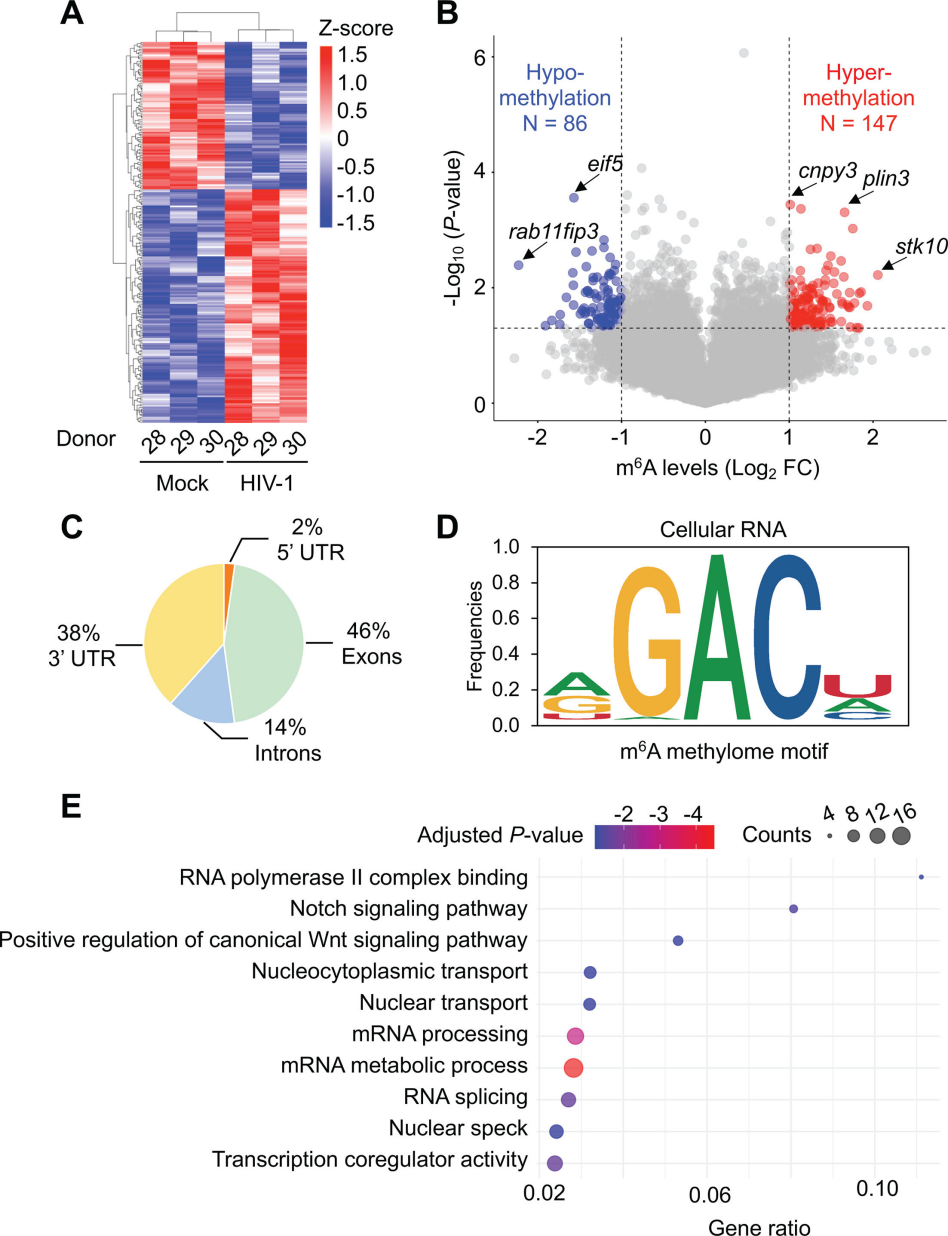
m^6^A-SAC-seq identifies cellular mRNAs that are differentially m^6^A-modified upon HIV-1 infection. Activated primary CD4^+^ T cells isolated from donor PBMCs were mock-infected or infected with HIV-1_NL4–3_ at an MOI of 1 for 96 h. Poly(A)-enriched RNA was used for m^6^A-SAC-seq. (**A**) Heat map showing transcript-level differences in m^6^A modification between mock and HIV-1-infected cultures. Due to the large data set, only genes with significant differences are displayed. Each row represents a m^6^A site, and each column represents a sample. Both rows and columns are clustered using correlation distance. (**B**) Volcano plot showing m^6^A-hypomethylated (blue) and m^6^A-hypermethylated (red) mRNA from HIV-1-infected cells compared with mock-infected controls. Adenosines that are considered differentially methylated in response to HIV-1 infection are ≥ 2-fold changed compared with mock-infected controls, with *P* < 0.05. (**C**) m^6^A distribution in different regions of cellular mRNA. Analysis was performed with mock and HIV-1-infected samples combined (*N* = 6). (**D**) m^6^A consensus motif frequencies in cellular RNA were determined using m^6^A-SAC-seq. (**E**) Gene ontology (GO) analysis of m^6^A-hypermethylated cellular genes in Metascape. The top 10 pathways with the lowest adjusted *P*-values were selected and visualized using a bubble chart generated by R. Gene ratio is the percentage of genes in each GO term that are differentially changed. Adjusted *P*-value = Benjamini-Hochberg adjusted *P*-value.

We created a heatmap to visualize and compare the m^6^A levels of cellular transcripts affected by HIV-1 infection (Fig. 2A). Overall, 86 m^6^A sites became hypomethylated (blue) and 147 sites became hypermethylated (red) during HIV-1 infection (Fig. 2B). Selected transcripts with a large log_2_ fold change (FC) or a high degree of significance are indicated by arrows. Analysis of the transcript-level distribution of m^6^A sites shows that exons contain a major portion of m^6^A sites (46%), followed by 3′ UTR (38%), introns (14%), and 5′ UTR (2%) (Fig. 2C). Analysis of the m^6^A motifs identified in cellular RNA reveals an internal consensus of GAC, with A/G/U and U/A/C in the terminal positions at the 5′ and 3′ ends of the motif, respectively (Fig. 2D). To identify the pathways related to cellular transcripts that are hypermethylated during HIV-1 infection, we conducted gene ontology (GO) pathway analysis (Fig. 2E). The analysis indicates that HIV-1 infection significantly regulates the m^6^A modification of transcripts involved in processes such as host RNA processing, signal transduction, and nucleocytoplasmic transport.

We also identified 30 m^6^A sites in HIV-1 RNA from infected primary CD4^+^ T cells (Fig. S2A; Table S2). The m^6^A distribution in HIV-1 open reading frames (ORFs) and the 3′ UTR is shown in Fig. S2B. Among these, the *pol* coding region was found to have the most m^6^A sites, with a total of 11. The site with the highest frequency of transcripts containing m^6^A modification is A8088, located in the sequence overlapping with the *env*, *rev, and tat* coding regions, with an average of 64.6% of transcripts having m^6^A modification among the three individual donors. We further analyzed the m^6^A consensus motifs in viral RNA and found that, like cellular RNA, the internal consensus sequence in viral RNA was GAC (Fig. S2C). However, m^6^A motifs in the viral RNA had a strong preference for A at the 5′ terminal position, in contrast to the more even distribution of A/G/U in cellular RNA m^6^A motifs. This preference may be a reflection of the A-richness of the HIV-1 genome compared with cellular RNA (18). Moreover, there is a preference for U at the 3′ terminal position of m^6^A motifs in cellular RNA (Fig. 2D) but not viral RNA (Fig. S2C). Overall, these data define the m^6^A epitranscriptomic landscape of cellular and viral RNA in HIV-1-infected primary CD4^+^ T cells.

**Fig 3:**
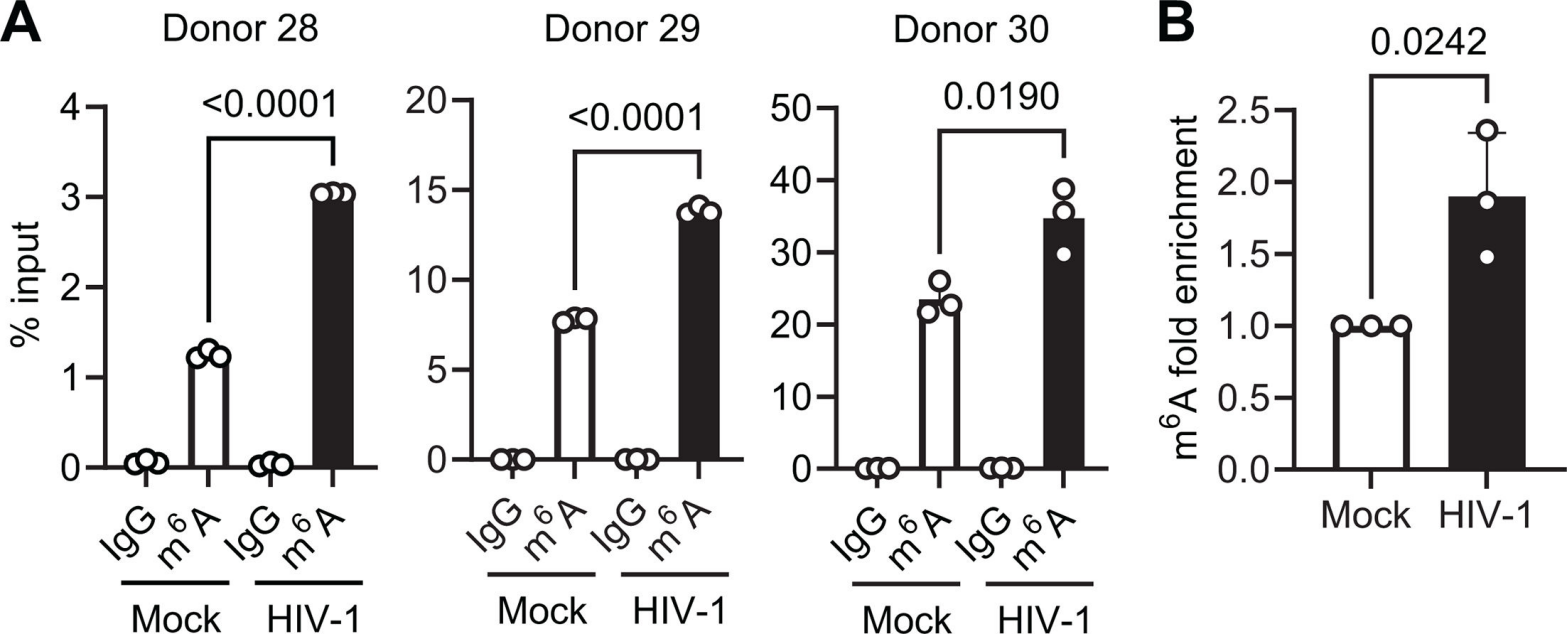
m^6^A modification of *PLIN3* mRNA is increased by HIV-1 infection in primary CD4^+^ T cells. Activated primary CD4^+^ T cells isolated from PBMCs of three donors were mock-infected or infected with HIV-1_NL4–3_ at an MOI of 1 for 96 h. Total cellular RNA was subjected to meRIP, and the level of m^6^A-modified transcripts in the meRIP was determined relative to (**A**) input or (**B**) mock-infected controls by RT-qPCR. Data are shown as mean ± SD. A two-tailed unpaired *t*-test was used for statistical analysis (*P* values are shown on the figures).

Table 1 lists the 10 cellular transcripts with the most significantly hypermethylated m^6^A sites located in an exon. We used meRIP and RT-qPCR to measure the relative overall m^6^A levels on the selected transcripts in HIV-1-infected Jurkat cells compared with mock-infected controls (Fig. S3). The results show that among these 10 genes, the m^6^A levels of disco interacting protein 2 homolog B (*DIP2B*), *PLIN3*, and MYC binding protein 2 (*MYCBP2*) showed a global increase in m^6^A levels in HIV-1-infected cells. meRIP and RT-qPCR were also used to confirm a significant increase in *PLIN3* mRNA m^6^A levels in HIV-1-infected primary CD4^+^ T cells compared with mock-infected controls (Fig. 3A and B). The observed hypermethylation of *PLIN3* mRNA was highly reproducible among primary cell donors (Table S1) and consistently ranked among the top hypermethylated transcripts with the smallest *P*-value (Table 1). Previous studies have identified PLIN3 as a regulator of HIV-1 replication (19–21). Therefore, we chose *PLIN3* mRNA for further functional studies.

**TABLE 1:** Top 10 hypermethylated m^6^A sites and their transcripts in HIV-1-infected primary CD4^+^ T cells^*a*^

Gene name (symbol)	log_2_ FC (HIV-1/Mock)	*P*-value	Key function
Disco interacting protein two homolog B (DIP2B)	1.76	0.0010	DNA methylation (22)
Hydroxyacyl-CoA dehydrogenase (HADH)	1.72	0.0219	Fatty acid oxidation (23)
MAX interactor 1 (MXI1)	1.71	0.0217	Antagonist of MYC (24)
Perilipin 3 (PLIN3)	1.66	0.0005	Lipid metabolism (25) and protein sorting (26)
MYC binding protein 2 (MYCBP2)	1.62	0.0034	E3 ligase (27)
Leukotriene A4 hydrolase (LTA4H)	1.57	0.0128	Cell cycle (28)
X-linked inhibitor of apoptosis (XIAP)	1.51	0.0393	Cell apoptosis (29)
ARF-like GTPase 14 effector protein (ARL14EP)	1.48	0.0216	Chromatin regulator (30)
E1A binding protein p300 (EP300)	1.43	0.0073	Transcriptional coactivator (31)
MALT1 paracaspase (MALT1)	1.42	0.0091	Lymphocyte activation (32)

^*a*^ Activated primary CD4^+^ cells from three different healthy donors were mock-infected or infected with HIV-1_NL4-3 _at an MOI of 1 for 96 h. Poly(A)-enriched RNA from cells was analyzed by m^6^A-SAC-seq. The top 10 cellular genes with significant m^6^A hypermethylation are listed in order of log_2 _fold change (FC) in m^6^A levels (*P* < 0.05). *PLIN3* shows the smallest *P*-value. All cellular transcripts analyzed by m^6^A-SAC-seq are included in Table S1 (Excel file).

### PLIN3 does not affect HIV-1 replication in Jurkat cells

Based on our findings that HIV-1 infection promotes m^6^A methylation of *PLIN3* mRNA in CD4^+^ T cells, we asked whether infection also changes PLIN3 expression in Jurkat CD4^+^ T cells. First, we used HIV-1 to infect Jurkat cells at an MOI of 1 for 72 h and then detected *PLIN3* mRNA and protein levels by RT-qPCR and immunoblotting, respectively. We found that HIV-1 infection did not change the steady-state level of *PLIN3* mRNA in Jurkat cells (Fig. S4A). Likewise, PLIN3 protein levels remain unchanged after HIV-1 infection of Jurkat cells (Fig. S4B and C).

We next aimed to determine whether PLIN3 regulates HIV-1 replication in Jurkat cells. To test this, we generated a *PLIN3* knockout (KO) Jurkat cell line by lentiviral transduction of vectors expressing Cas9 and *PLIN3-*specific single-guide RNA (sgRNA). Jurkat cells transduced with a scrambled control sgRNA were used as a control (Ctrl). Ctrl and *PLIN3* KO cells were infected with HIV-1 at an MOI of 1 for 72 h. Immunoblotting was used to determine the expression levels of PLIN3 and HIV-1 proteins (Fig. S4D). As expected, no PLIN3 expression was observed in *PLIN3* KO cells (Fig. S4D), and there was no difference in PLIN3 expression in Ctrl cells after HIV-1 infection (Fig. S4B and D). Furthermore, we observed no significant difference in the expression of HIV-1 Env, Gag, or CA in the absence of PLIN3 expression (Fig. S4D and E). We measured HIV-1 p24 release in cell culture supernatants and found no difference between the Ctrl and *PLIN3* KO cells, suggesting that PLIN3 is not required for virion release (Fig. S4F). Finally, virus input was normalized by p24 content prior to infection of TZM-bl luciferase indicator cells to measure viral infectivity. The results confirmed that the infectivity of HIV-1 virions produced in *PLIN3* KO cells was not different from that in Ctrl cells (Fig. S4G). Overall, our results demonstrate that HIV-1 infection does not alter the RNA or protein levels of PLIN3, and PLIN3 KO does not change HIV-1 infection in Jurkat CD4^+^ T cells. These results are consistent with a previous report of PLIN3 and HIV-1 infection in Jurkat and HeLa cells (33).

### HIV-1 infection increases *PLIN3* mRNA levels but does not alter *PLIN3* mRNA stability in primary CD4^+^ T cells

**Fig 4:**
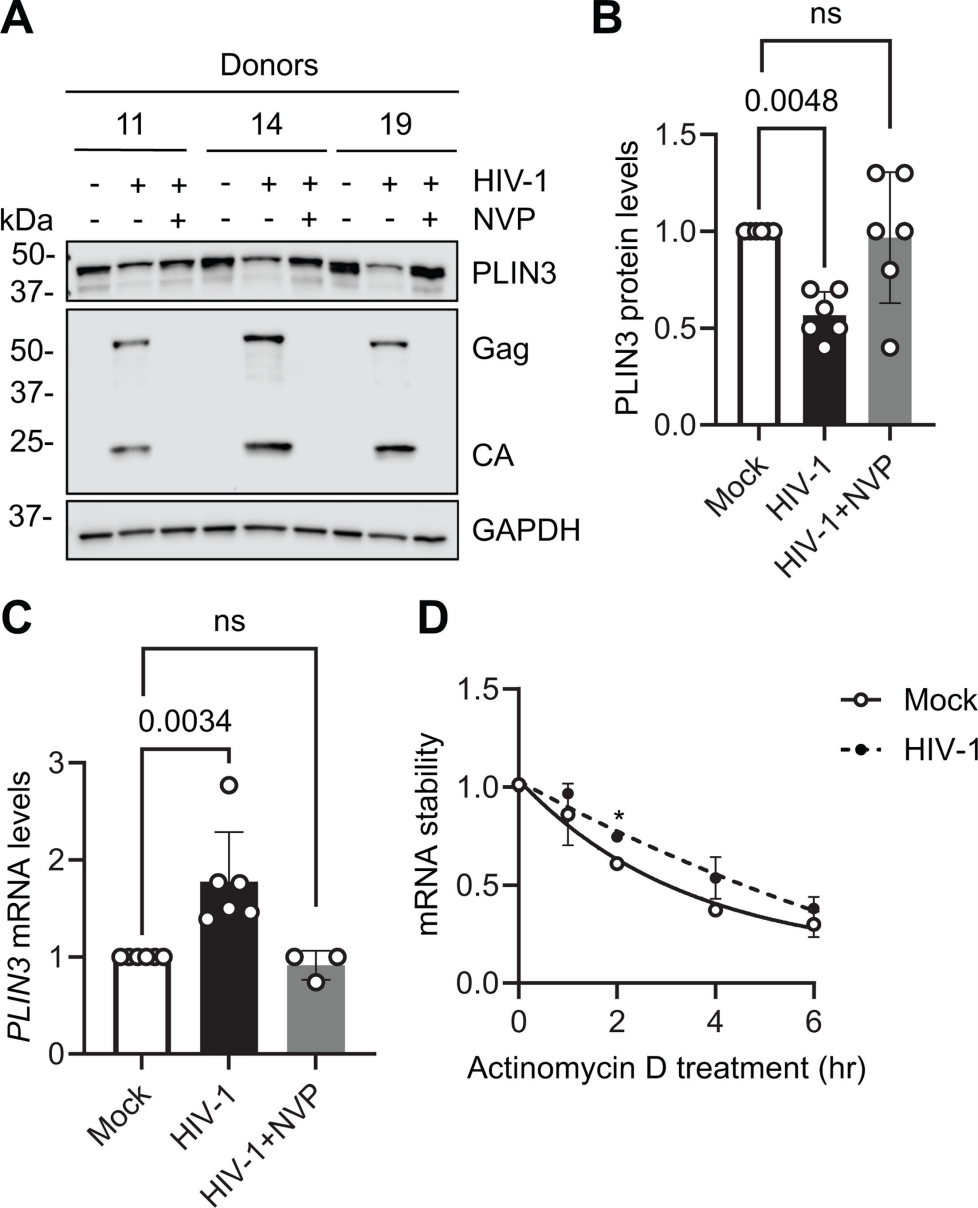
HIV-1 infection increases *PLIN3* mRNA levels but decreases PLIN3 protein levels in primary CD4^+^ T cells. (**A–D**) Primary CD4^+^ T cells were mock-infected or infected with HIV-1_NL4–3_ at an MOI of 1 for 96 h. The HIV-1 reverse transcription inhibitor NVP was used to block viral replication (HIV-1 + NVP). GAPDH was used as a loading control. (**A**) PLIN3 and HIV-1 protein expression were measured by IB. A representative IB is shown. (**B**) Relative levels of PLIN3 protein expression normalized with GAPDH, as shown in (**A**), from three individual donors. (**C**) PLIN3 mRNA levels were measured by RT-qPCR. *N* = 6 (Mock, HIV-1) or *N* = 3 (HIV-1 + NVP). (**D**) Cells were treated with actinomycin D at 96 hpi. Samples were collected at the indicated time points, and *PLIN3* mRNA levels were detected by RT-qPCR. Data are shown as means ± SD of results from three donors’ cells (raw data in Fig. S5A). Ordinary one-way ANOVA with Dunnett correction (B, and C) and multiple unpaired *t*-test (**D**) were used for statistical analysis (*P* values are shown on figures). ns, not significant. * *P* < 0.05.

We next sought to determine whether HIV-1 infection alters the RNA or protein levels of *PLIN3* in primary CD4^+^ T cells. Cells from three individual donors were mock-infected or infected with HIV-1 at an MOI of 1 for 96 h. To block HIV-1 replication, we used the reverse transcription inhibitor nevirapine (NVP) as a control. HIV-1 Gag and CA were detected to confirm infection. Immunoblotting showed that the level of PLIN3 was significantly decreased by HIV-1 infection (Fig. 4A and B). In contrast, the levels of *PLIN3* mRNA were increased by HIV-1 infection (Fig. 4C).

Given that m^6^A can regulate mRNA levels by altering mRNA stability (34, 35), we examined *PLIN3* mRNA stability in mock and HIV-1-infected primary CD4^+^ T cells. Cells from six individual donors were mock-infected or infected with HIV-1 at an MOI of 1 for 96 h (Fig. S5A) or 90 h (Fig. S5B). Actinomycin D was then added to the culture medium to inhibit transcription, and samples were collected at the indicated times to measure the relative levels of *PLIN3* mRNA. The average results from three donors’ cells indicate that the stability of *PLIN3* mRNA may be slightly increased in HIV-1-infected cells compared with mock at 96 hpi; however, this effect was only statistically significant at 2 h post-actinomycin D treatment (Fig. 4D). When cells were infected with HIV-1 for 90 h prior to actinomycin D treatment, no differences in *PLIN3* mRNA stability were observed (Fig. S5B). Together, these results suggest that the increase in *PLIN3* mRNA levels observed upon infection of primary CD4^+^ T cells is not due to increased stability of the transcript.

### HIV-1 infection increases the levels of *PLIN3* mRNA in the nucleus of primary CD4^+^ T cells

**Fig 5:**
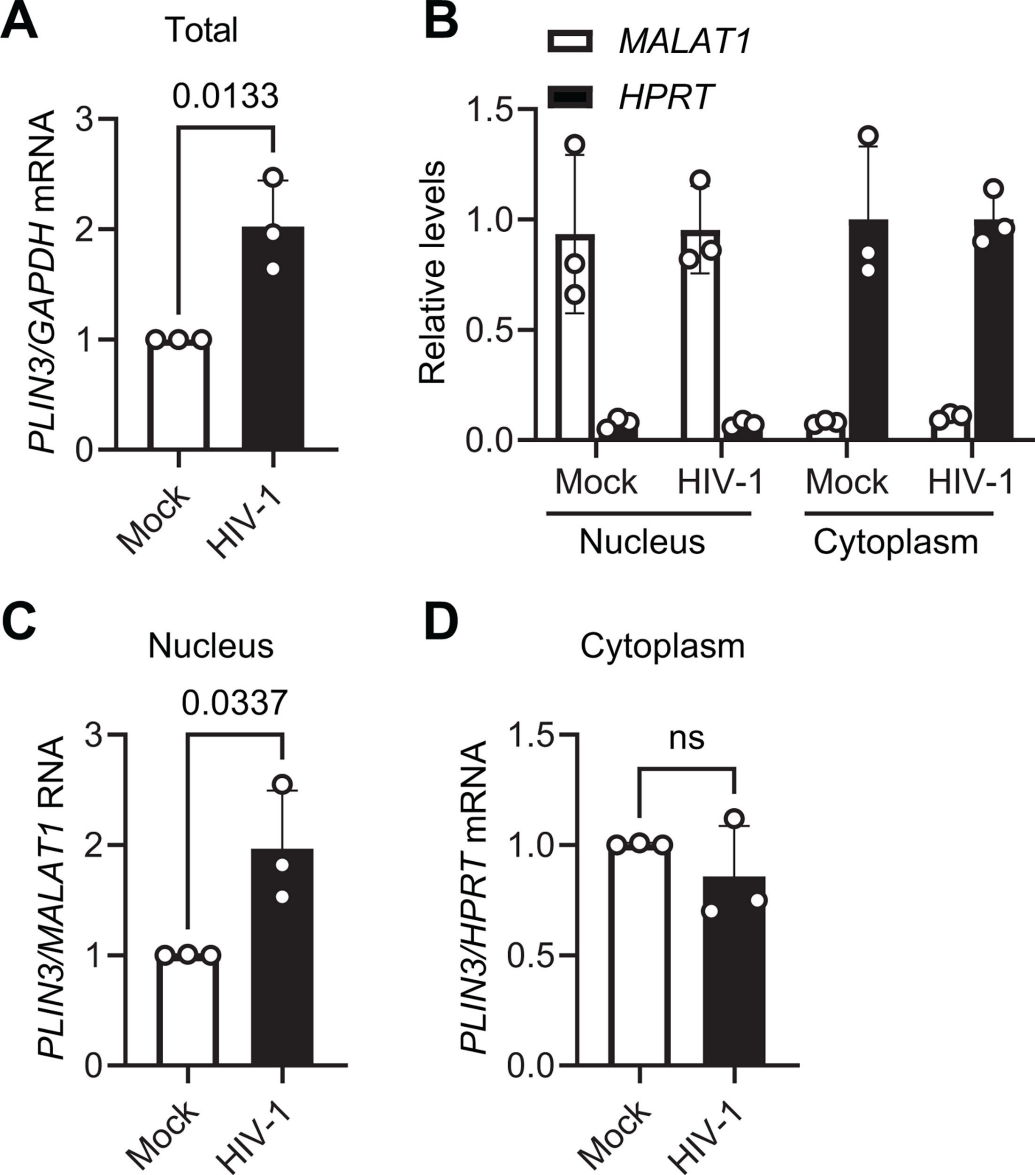
HIV-1 infection increases the levels of *PLIN3* mRNA in the nucleus of primary CD4^+^ T cells. (**A–D**) Activated primary CD4^+^ T cells from three healthy donors were mock-infected or infected with HIV-1_NL4–3_ at an MOI of 1 for 96 h. Each dot represents the result from one donor’s cells. Data are shown as means ± SD. (**A**) *PLIN3* mRNA levels in total cell lysates were measured by RT-qPCR and normalized with GAPDH. (**B**) *MALAT1* lncRNA and *HPRT* mRNA levels from each fraction were measured by RT-qPCR to confirm successful separation of the nucleus and cytoplasm, respectively. (**C and D**) Cellular RNA was separated into nuclear and cytoplasmic fractions prior to RT-qPCR analysis. *PLIN3* mRNA levels from the nuclear and cytoplasmic fractions are shown relative to *MALAT1* and *HPRT*, respectively. A two-tailed unpaired *t*-test was used for statistical analysis. *P* value is shown on the figure. ns, not significant.

We next sought to determine whether HIV-1 infection influences the nucleocytoplasmic localization of *PLIN3* mRNA. Consistent with results in Fig. 4C, HIV-1 infection of primary CD4^+^ T cells from three additional donors significantly upregulated *PLIN3* mRNA levels in whole cell lysates (Fig. 5A). Nucleocytoplasmic fractionation of mock or HIV-1-infected primary CD4^+^ T cell lysates was performed, and *PLIN3* mRNA levels were measured in the nuclear and cytoplasmic fractions (Fig. 5B through D). Successful fractionation was demonstrated by measuring RNA markers for the nucleus and cytoplasm, respectively. Metastasis-associated lung adenocarcinoma transcript 1 (*MALAT1*) is a nuclear long non-coding RNA (lncRNA), whereas hypoxanthine-guanine phosphoribosyltransferase 1 gene (*HPRT*) mRNA was found in the cytoplasm (Fig. 5B). Interestingly, *PLIN3* mRNA accumulated more in the nucleus during HIV-1 infection of primary CD4^+^ T cells compared with mock controls (Fig. 5C), whereas the levels of *PLIN3* mRNA in the cytoplasm were not significantly different (Fig. 5D). These results help explain the observed total increase of *PLIN3* mRNA in infected cells compared with mock controls (Fig. 4C and 5A).

### *PLIN3* mRNA is less actively translated during HIV-1 infection of primary CD4^+^ T cells

**Fig 6:**
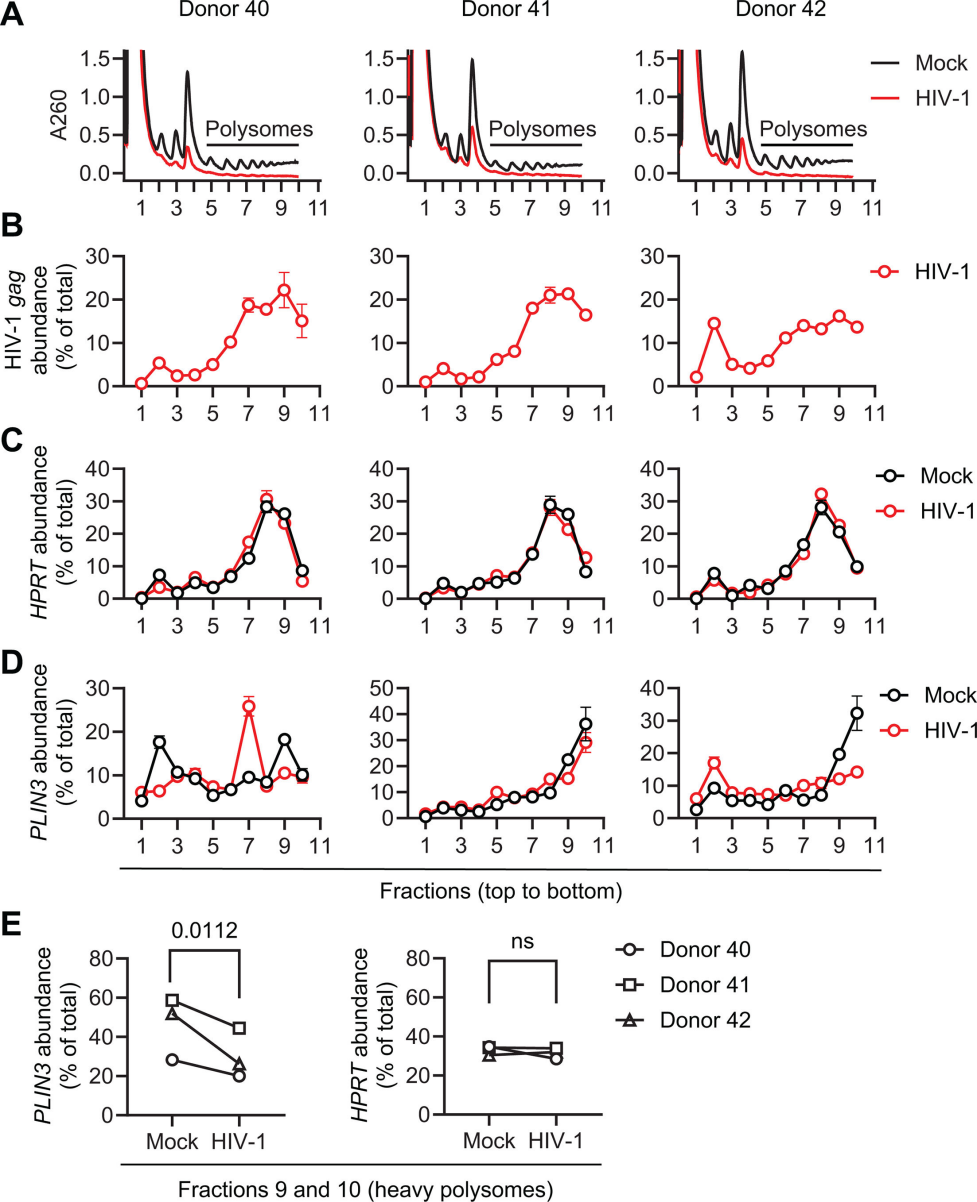
Polysome profile analysis of HIV-1-infected primary CD4^+^ T cells from three donors. (**A–E**) Activated primary CD4^+^ T cells from three healthy donors were mock-infected or infected with HIV-1_NL4–3_ at an MOI of 1 for 96 h. (**A**) Polysome profile analysis (fractions 1-10, from top to bottom of the sucrose gradient). (**B–D**) Relative mRNA levels of (**B**) HIV-1 *gag,* (**C**) *HPRT,* and (**D**) *PLIN3* from each fraction were measured by RT-qPCR. (**E**) Relative abundance of *PLIN3* and *HPRT* in combined fractions 9 and 10 was measured by RT-qPCR. A two-tailed unpaired *t*-test was used for statistical analysis. *P* value is shown on the figure. ns, not significant. (**B–E**) The total detected quantity of a transcript in all fractions is set to 100% and the proportion of transcript found in each fraction (**B–D**) or combined two fractions (**E**) is represented as a percentage.

Despite equal levels of *PLIN3* mRNA in the cytoplasm of mock and HIV-1-infected primary CD4^+^ T cells (Fig. 5D), PLIN3 protein was significantly reduced in infected cells (Fig. 4A and B). We therefore sought to determine whether HIV-1 infection reduces the translation of *PLIN3* mRNA. Primary CD4^+^ T cells from three individual donors were mock-infected or infected with HIV-1_NL4-3_ at an MOI of 1 for 96 h. Translation was stalled using an acute treatment of cycloheximide, and polysomes were prepared by fractionation of sucrose gradients (36). The polysome profiles of mock and infected-cell lysates demonstrated successful fractionation (Fig. 6A). However, it should be noted that despite using an equal number of cells, infected cells had lower overall ribosome content, as indicated by the A_260_ absorbance values (Fig. 6A). This might be due to HIV-1 Vpr-mediated impairment of cellular protein translation as previously reported (37). HIV-1 infection was confirmed by detection of p24 in the cell culture supernatants (Fig. S6) and detection of the full-length viral *gag* RNA in polysome-containing fractions (Fig. 6B). As expected, we found no difference in the amount of *HPRT* mRNA associated with polysomes when cells were infected with HIV-1 compared with mock (Fig. 6C and E). In contrast, we found that in HIV-1-infected cells, *PLIN3* mRNA was less associated with the highly translated heavy polysomes in fractions 9 and 10 (Fig. 6D and E). Together with the observed similar steady state levels of *PLIN3* mRNA in the cytoplasm (Fig. 5D), these results provide evidence that *PLIN3* mRNA is translated less efficiently in HIV-1-infected primary CD4^+^ T cells compared with mock controls.

### Knockdown of PLIN3 in primary CD4^+^ T cells decreases HIV-1 production but increases viral infectivity

**Fig 7:**
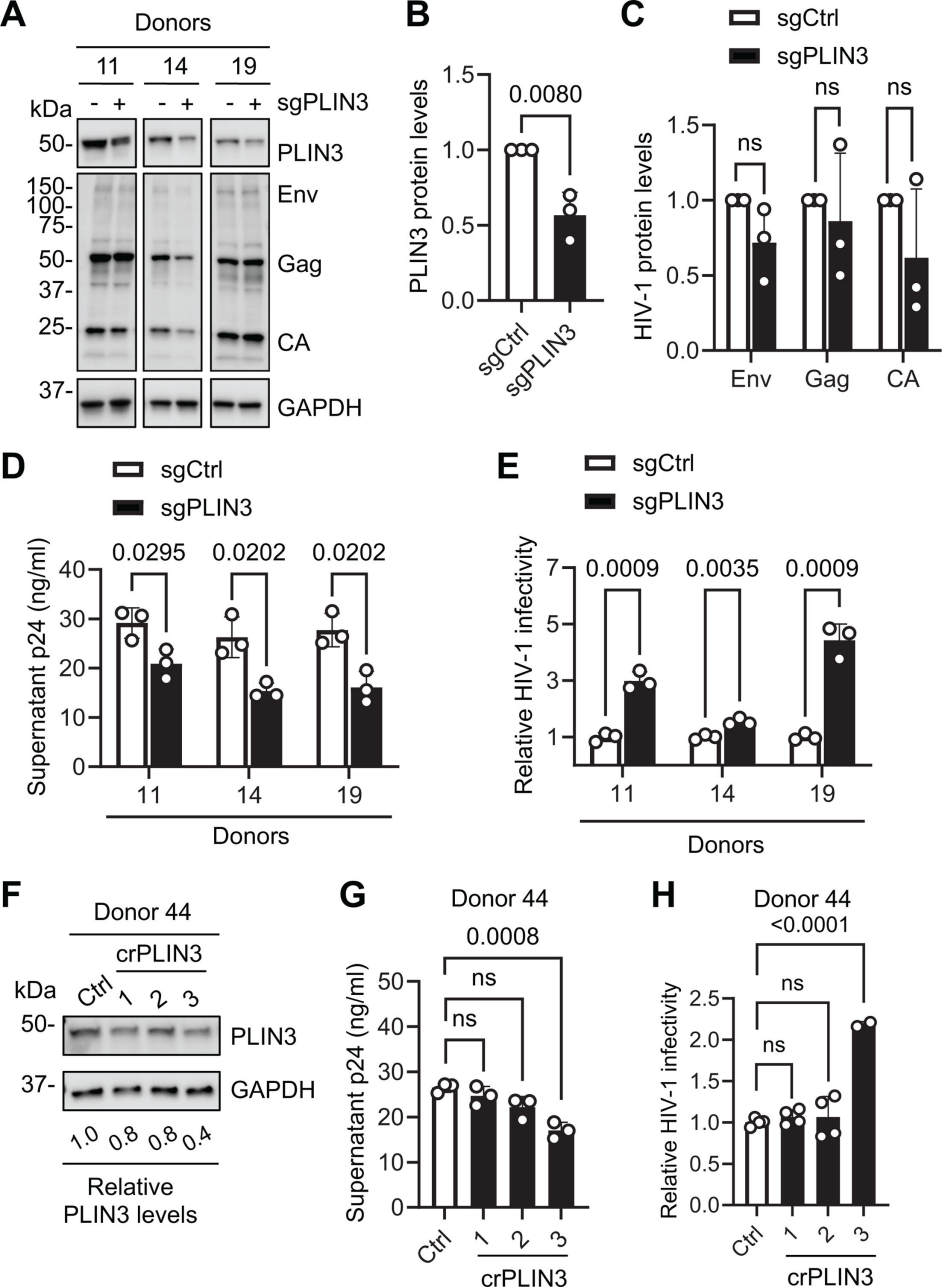
Knockdown of PLIN3 in primary CD4^+^ T cells decreases HIV-1 production but increases viral infectivity. (**A–D**) Primary CD4^+^ T cells were transduced with lentiviral vectors expressing non-targeting (Ctrl) or PLIN3 small guide (sg) RNA to achieve partial stable knockdown of PLIN3. Cells were then infected with HIV-1_NL4–3_ at an MOI of 1 for 96 h. (**A**) Relative levels of PLIN3 expression and HIV-1 infection were measured by IB in cells from three independent donors. (**B**) Relative quantification of PLIN3 protein expression is shown in (**A**). (**C**) Relative levels of HIV-1 protein expression are shown in (**A**). (**D**) Cell supernatant p24 levels from HIV-1-infected cells were quantified by ELISA. (**E**) TZM-bl cells were infected with HIV-1 collected from sgCtrl or sgPLIN3 cell culture supernatants. Luciferase activity was measured at 48 hpi. (**F–H**) Primary activated CD4^+^ T cells from one additional donor were nucleofected with control crRNA or one of three different crRNAs specifically targeting *PLIN3*. (**F**) Western blot analysis of PLIN3 knockdown efficiency. GAPDH was used as a loading control. Relative PLIN3 levels were normalized to GAPDH. (**G**) The cells used in (**F**) were subsequently infected with HIV-1_NL4-3_ at an MOI of 1 for 96 h, and p24 levels in the supernatant were measured by ELISA. (**H**) TZM-bl cells were infected with HIV-1 collected from Ctrl or crPLIN3 cell culture supernatants. Luciferase activity was measured at 48 hpi and normalized to protein amounts. Data are shown as means ± SD from three individual donors or 2–4 technical replicates. A two-tailed unpaired *t*-test (**B**), multiple unpaired *t*-test (**C–E**), and one-way ANOVA (**G and H**) were used for statistical analysis (*P* values are shown on figures). ns, not significant.

We next sought to investigate whether PLIN3 is necessary for HIV-1 infection in primary CD4^+^ T cells. Cells were transduced with lentiviral vectors expressing Cas9 and a scramble control (sgCtrl) or *PLIN3-*specific sgRNA (sgPLIN3), followed by infection with HIV-1 at an MOI of 1 for 96 h. Immunoblot analysis indicated that PLIN3 levels were reduced by ~2-fold in cells from all three donors (Fig. 7A and B). PLIN3 knockdown did not significantly affect the levels of cell-associated Env, Gag, or CA (Fig. 7A and C). Furthermore, HIV-1 p24 levels in the cell culture supernatants were reduced in sgPLIN3 cells compared with sgCtrl cells (Fig. 7D). Virus input was normalized by p24 content prior to infection of TZM-bl cells to measure viral infectivity. The results showed that the infectivity of HIV-1 produced by sgPLIN3 cells was significantly increased compared with viruses produced by sgCtrl cells (Fig. 7E). To better silence PLIN3 protein expression and validate the above results, we further used nucleofection to deliver three independent CRISPR RNAs (crRNAs) targeting PLIN3 (crPLIN3-1, -2, or -3) into primary activated CD4^+^ T cells from three additional donors and showed representative results from one donor. Immunoblot analysis confirmed that crPLIN3-3 nucleofection effectively reduced PLIN3 protein levels by 60% compared with control cells, whereas crPLIN3-1 and crPLIN3-2 only reduced protein levels by 20% (Fig. 7F). Subsequent infection with HIV-1 revealed that primary CD4^+^ T cells nucleofected with crPLIN3-3 produced significantly lower levels of HIV-1 p24 (*P* = 0.0008) in the supernatants compared with control cells (Fig. 7G). Furthermore, viral infectivity of HIV-1 released from crPLIN3-3 nucleofected cells was significantly higher (*P* < 0.0001) than HIV-1 released from control cells (Fig. 7H). Together, these results suggest that PLIN3 positively regulates HIV-1 release but inversely affects viral infectivity in primary CD4^+^ T cells.

### Mutation of cytosine in the m^6^A DRACH motif in exon 8 of *PLIN3* mRNA inhibits m^6^A modification

We next sought to determine the functional significance of individual m^6^A sites in the *PLIN3* mRNA. Our m^6^A-SAC-seq analysis revealed that the relative abundance of m^6^A changed significantly at three sites upon HIV-1 infection of primary CD4^+^ T cells (Table S1). One of these three sites (A1218) is in the last exon of the ORF (exon 8), whereas the two other sites (A1396 and A2072) are in the 3′ UTR. Of note, m^6^A modification of the site in exon 8 (A1218) increases significantly in HIV-1-infected cells, whereas each site in the 3′ UTR (A1396 or A2072) exhibits decreased m^6^A modification (Table S1). Mutation of the adenosine (A1218) in exon eight unavoidably changes the amino acid sequence of PLIN3, making data interpretation difficult. Therefore, we sought to develop an alternate method for preventing m^6^A modification in a site-specific manner. As an alternative approach, we mutated the conserved cytosine of the m^6^A DRACH motif (1219C > T) in expression plasmids containing the PLIN3 coding sequence (Fig. S7A). Of note, this mutation largely preserves the predicted RNA structure and thermodynamic stability of *PLIN3* mRNA without changing the protein coding sequence (Fig. S7A).

HEK293T cells were transfected with WT or 1219C > T plasmids, and m^6^A levels were measured using a single-base elongation- and ligation-based qPCR amplification method (SELECT) (38). As controls, 28S rRNA contains a single m^6^A modification at nucleotide 4190, whereas the adenosine at position 4194 remains unmethylated (39). Therefore, we detected modification levels at these sites as negative (A4194) and positive controls (m^6^A4190) for the SELECT assay and obtained the expected results (Fig. S7B). Next, we measured the relative levels of m^6^A in the *PLIN3* mRNA at nucleotide position 248 in exon 3 and position 1218 in exon 8, adjacent to the 1219C > T mutation. As expected, we observed no difference in m^6^A levels at site 248 in cells transfected with the *PLIN3* 1219C > T plasmid compared with wild type (WT). In contrast, there was a significant decrease in modification of m^6^A1218 when the adjacent cytosine in the DRACH motif was mutated (1219C > T) (Fig. S7C). This result suggests that mutation of the DRACH cytosine of exon 8 of the *PLIN3* mRNA inhibits the installation of m^6^A.

### Mutation of cytosine in the m^6^A DRACH motif in exon 8 of *PLIN3* mRNA reduces mRNA levels

To determine whether the 1219C > T mutation in exon 8 of PLIN3 affects mRNA stability, HEK293T cells were transfected with WT or 1219C > T plasmids for 48 h and then treated with actinomycin D to inhibit transcription. The results show no significant difference between WT and mutant PLIN3 1219C > T (Fig. S8A). To examine the nucleocytoplasmic localization of *PLIN3* mRNA, we performed the fractionation of transfected HEK293T cells and measured *PLIN3* mRNA levels in nuclear and cytoplasmic fractions. Successful fractionation was demonstrated by measuring *MALAT1* in the nucleus and *HPRT* mRNA in the cytoplasm (Fig. S8B). Interestingly, the level of *PLIN3* 1219C > T mRNA in whole cells or in the nucleus was significantly lower than WT, whereas this difference was not observed in the cytoplasmic fraction (Fig. S8C). These data suggest that m^6^A modification of *PLIN3* at the 1218 site regulates the expression and localization of *PLIN3* mRNA.

## DISCUSSION

m^6^A modification of HIV-1 RNA and the proteins involved in deposition, recognition, and removal of m^6^A marks play important roles in HIV-1 replication by regulating RNA stability, alternative splicing, RNA packaging, and Gag synthesis (40–44). In this study, we sought to identify cellular transcripts that are differentially m^6^A-modified upon HIV-1 infection to provide novel insight into how HIV-1 regulates host gene expression.

It is well-established that HIV-1 infection causes an upregulation of cellular RNA m^6^A levels in a variety of cell types (8–12). Our previous study showed that HIV-1 envelope proteins upregulate m^6^A levels of cellular RNA independently of viral replication in CD4^+^ T cells (8). These findings suggest that HIV-1 may exploit the host m^6^A machinery to modulate viral infection. Alternatively, the increase in m^6^A may be a general host cell response to HIV-1 infection. Regardless, how m^6^A upregulation occurs during HIV-1 infection remains unclear. Our previous results showed that the expression levels of m^6^A writers and erasers were not altered by HIV-1 infection in primary CD4^+^ T cells or latently infected cells after reactivation (8, 12). The absence of changes in writer or eraser protein levels suggests that the upregulation of m^6^A may result from an increase in methyltransferase activity rather than protein expression. Therefore, we conducted co-IP to examine the level of interaction between METTL3 and 14 during HIV-1 infection compared with uninfected controls. We found that HIV-1 infection enhances the METTL3/14 interaction in CD4^+^ T cells (Fig. 1C and F). The increased interaction could potentially be attributed to post-translational modifications of METTL3 or METTL14, which can regulate enzyme activity and influence the overall dynamics of m^6^A regulation (45–47).

Previously reported m^6^A sequencing of RNA from HIV-1-infected cells using meRIP-seq and CLIP-seq has provided insight into the general location of mRNA m^6^A modifications (48–50). However, two major disadvantages of meRIP-seq and CLIP-seq are low resolution and the lack of m^6^A/A quantification. In this study, we employed m^6^A-SAC-seq to quantitatively identify individual m^6^A modifications on a transcriptome-wide scale in both cellular and HIV-1 RNA (16, 17). These data are the first to report how productive HIV-1 infection regulates m^6^A modification, at single-base resolution, in primary CD4^+^ T cells and provide a foundation for targeted functional studies.

For the purposes of the current study, we chose to focus on transcripts that become significantly hypermethylated in primary CD4^+^ T cells upon HIV-1 infection compared with mock-infected controls. Our GO pathway analysis of these transcripts found an association between HIV-1 infection and mRNA splicing (Fig. 2E), which is consistent with a previous study that performed gene set enrichment analysis (GSEA) of m^6^A sequencing from HIV-1-infected hippocampus from a transgenic rat (51). This suggests that m^6^A modification of host cell RNA may be a regulatory mechanism of gene expression that affects RNA splicing during HIV-1 infection.

We identified a total of 30 m^6^A modifications in HIV-1 RNA, which is fewer than our previous analysis of RNA from J-Lat cells grown under conditions of latency reactivation. It is possible that HIV-1 RNA expressed after latency reactivation is more heavily m^6^A-modified than transcripts made during productive infection (12). However, given the overlap between these two data sets (22 out of 30 m^6^A sites), it is more likely that this difference reflects the much lower percentage of primary cells expressing HIV-1 transcripts (data not shown). Of the eight m^6^A sites unique to the current study, seven were present on less than 10% of transcripts (Table S2). However, site A8660 in the *nef* region was modified in 58.3% transcripts, making this an interesting m^6^A modification for further study. Consistent with our previous study, three high-frequency modifications were present at A8088, A8984, and A8998 (Fig. S2A; Table S2). These three m^6^A sites were also identified by direct RNA sequencing in both HIV-1 producer HEK293T cells and infected CD4^+^ T cells and were implicated in viral RNA splicing (12, 41).

We chose to focus further on m^6^A modification of *PLIN3* mRNA during HIV-1 infection of primary CD4^+^ T cells. PLIN3, also known as TIP47 (Tail-interacting protein of 47 kDa), is a cellular protein that plays a crucial role in lipid droplet formation (25) and protein sorting (26). It has been demonstrated that lipid rafts are important for the replication of many viruses in multiple cell types (52). Particularly, the interaction of Gag with plasma membrane rafts plays a critical role in HIV-1 assembly and release HIV-1 (53). The current study is the first to reveal the effects of HIV-1 infection on *PLIN3* mRNA methylation and expression levels in primary CD4^+^ T cells. HIV-1 infection increased PLIN3 m^6^A methylation and resulted in an upregulation of total *PLIN3* mRNA levels (Fig. 3A, B, and 4C). Nucleocytoplasmic fractionation of infected-cell lysates showed an increase in nuclear *PLIN3* mRNA, whereas the levels of *PLIN3* mRNA in the cytoplasm remained unchanged compared with mock controls (Fig. 5). These results suggest a decrease in *PLIN3* mRNA export in HIV-1-infected primary CD4^+^ T cells.

Despite the increase in mRNA levels, the level of PLIN3 protein was significantly decreased by HIV-1 infection (Fig. 4A and B). We did not observe a significant difference in *PLIN3* mRNA stability in HIV-1-infected cells compared with mock controls (Fig. 4D and 5). However, polysome fractionation and measurement of polysome-associated RNA revealed that *PLIN3* mRNA is less efficiently translated in HIV-1-infected cells, despite similar amounts of cytoplasmic transcript available for engaging with ribosomes (Fig. 6). The results of our transfection-based assays in HEK293T cells suggest that the m^6^A sites identified by m^6^A-SAC-seq in the *PLIN3* mRNA are functional and can influence nuclear retention or translational efficiency (Fig. S7 and S8). Future studies will determine whether the same is true in primary CD4^+^ T cells.

Several previous studies explored the function of PLIN3 during HIV-1 infection. One study reported that HIV-1 Env binds to PLIN3 to target the trans-Golgi network in HeLa cells, which is essential for the efficient incorporation of HIV-1 Env into virions (19). Subsequent studies built upon these observations and showed that PLIN3 interacts with HIV-1 Gag and Env (20) and is essential to produce infectious HIV-1 in both Jurkat T cells and primary macrophages (20, 21). However, one group reevaluated the role of PLIN3 in HIV-1-infected Jurkat cells and found that PLIN3 was dispensable for Env virion incorporation (33). However, whether PLIN3 plays a role in HIV-1 infection of primary CD4^+^ T cells has not been reported. In this study, we demonstrated that knockdown of PLIN3 in primary CD4^+^ T cells reduced HIV-1 virion release but increased virion infectivity (Fig. 7). Since activated CD4^+^ T cells are the primary target of HIV-1 *in vivo*, these results are likely a more accurate reflection of how PLIN3 interacts with HIV-1 in the physiological environment. Future studies will focus on the role of PLIN3 in HIV-1 Env incorporation to better understand how PLIN3 regulates HIV-1 infection in primary CD4^+^ T cells.

**Fig 8:**
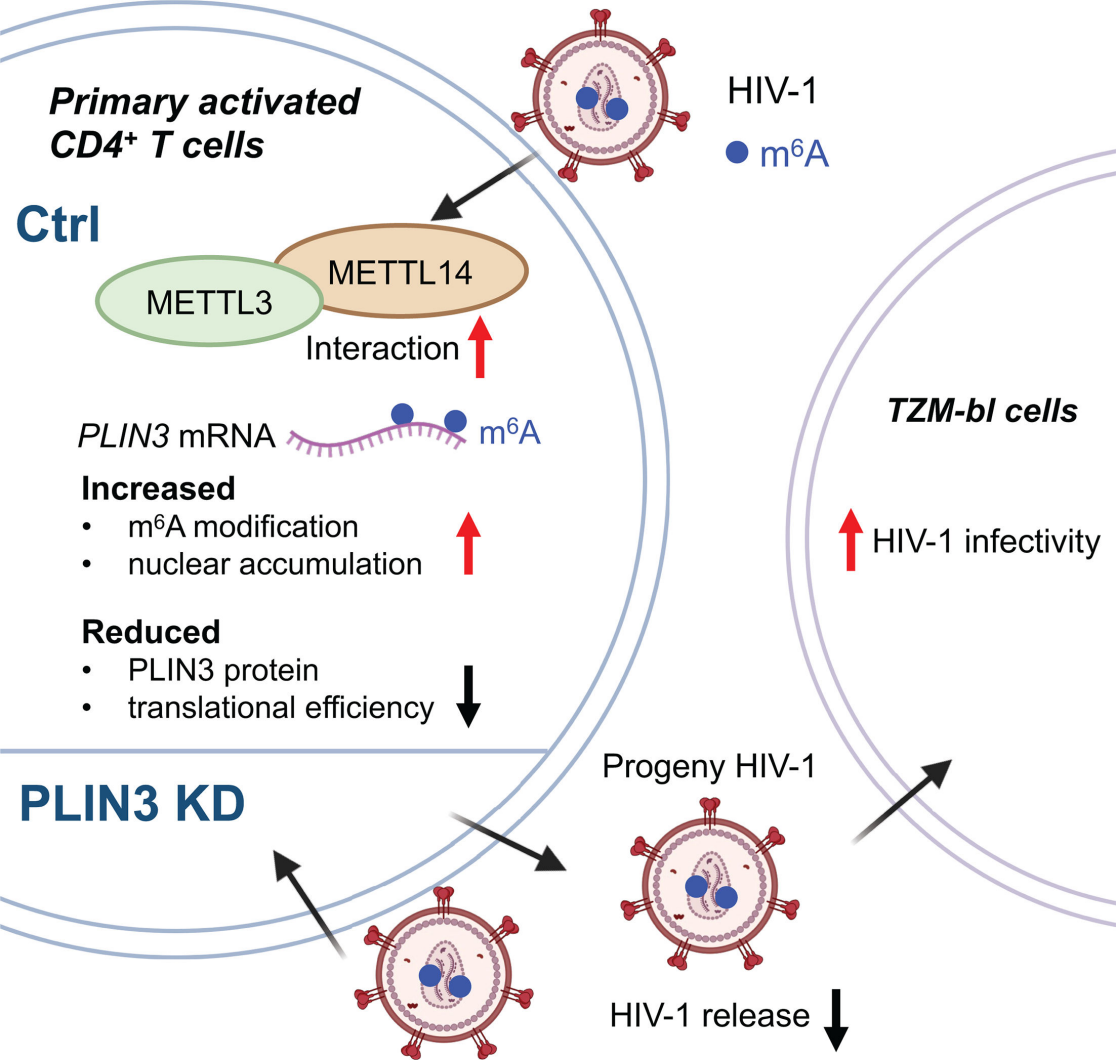
Summary and proposed model. In primary CD4^+^ T cells, HIV-1 infection promotes the interaction between METTL3 and METTL14. HIV-1 infection increases m^6^A level and nuclear accumulation of *PLIN3* mRNA but reduces PLIN3 protein expression and translation efficiency. Knockdown of PLIN3 in primary CD4^+^ T cells decreases HIV-1 release but increases viral infectivity in TZM-bl cells. Ctrl, control; KD, knockdown.

In summary, we identified m^6^A modification sites at single-base resolution on viral and cellular RNA that occur in response to HIV-1 infection of primary CD4^+^ T cells. We observed that HIV-1 infection promotes the interaction between METTL3/METTL14. HIV-1 infection of primary CD4^+^ T cells increases the m^6^A levels and nuclear accumulation of *PLIN3* mRNA but decreases translation of the message in the cytoplasm. We also demonstrate that PLIN3 influences HIV-1 release and infectivity in primary CD4^+^ T cells (Fig. 8). These findings highlight the critical role of m^6^A modification in regulating host gene expression and HIV-1 replication in primary CD4^+^ T cells.

## MATERIALS AND METHODS

### Cell culture

Jurkat and primary CD4^+^ T cells were cultured in RPMI-1640 (ATCC) supplemented with 10% fetal bovine serum (FBS; R&D Systems) and antibiotics (100 U/mL penicillin and 100 µg/mL streptomycin, Gibco). HEK293T, Ghost/X4/R5, and TZM-bl cells were cultured in DMEM (Gibco) with 10% FBS and antibiotics (8). All cells were cultured at 37°C with 5% CO_2_ and tested negative for mycoplasma contamination using a PCR-based universal mycoplasma detection kit (ATCC 30-1012K). Healthy deidentified donor blood was purchased from the DeGowin Blood Center at the University of Iowa. PBMCs were isolated from healthy donor blood using Ficoll-Paque PLUS (17144002, Cytiva). CD4^+^ T cells were enriched using EasySep Human CD4^+^ T cell isolation kit (17952, STEMCELL Technologies) and activated using ImmunoCult Human CD3/CD28/CD2 T cell activator (10970, STEMCELL Technologies) for 72 h.

### HIV-1 production and infection

Replication-competent X4-tropic HIV-1_NL4-3_ and R5-tropic HIV-1_NLAD8_ stocks were generated by transfection of HEK293T cells with pNL4-3 or pNLAD8 using jetPRIME (114-07, Polyplus Transfection) as described (8, 54). The supernatants were filtered (0.45 µm) and digested with DNase I (Turbo, Invitrogen) for 30 min at 37°C. The viral stock titers were calculated through serial dilution on Ghost/X4/R5 cell lines. For HIV-1 infection, Jurkat and primary activated CD4^+^ T cells were infected with HIV-1 at an MOI of 1. Spinoculation was performed by centrifuging the cells with the virus at 1,200 × *g* for 2 h at 25°C. Cells were washed twice with Dulbecco’s phosphate-buffered saline (DPBS) and resuspended in a fresh culture medium. The reverse transcriptase inhibitor NVP (10 µM, 4666, the AIDS Research and Reference Reagent Program, NIH) was used as a negative control. HIV-1 supernatant p24 levels were detected by p24 ELISA using anti-p24-coated plates (AIDS and Cancer Virus Program, National Cancer Institute, Frederick, MD) as described (55).

### Antibodies and immunoblotting

Antibodies used for immunoblotting were as follows: HIV-1 p24 (clone #24-2, the AIDS Research and Reference Reagent Program, NIH), GAPDH (AHP1628, Bio-Rad), METTL3 (15073, Proteintech), METTL14 (CL4252, Abcam), PLIN3 (10694-1-AP, Proteintech), and HIV-Ig (3957, the AIDS Research and Reference Reagent Program, NIH). Cells were harvested and lysed in cell lysis buffer (9803, Cell Signaling Technology) with a protease and phosphatase inhibitor (A32959, Pierce, Thermo Scientific). Immunoblotting was performed as described (56). GAPDH was used as a loading control for all immunoblots.

### RNA isolation and poly(A) enrichment

Total RNA was extracted using TRIzol (Invitrogen), and the RNA concentrations were determined by Nanodrop. mRNA was enriched using Dynabeads oligo(dT)25 (61005, Invitrogen) following the manufacturer’s instructions.

### m^6^A ELISA

m^6^A levels were quantified in 50 ng mRNA using an m^6^A RNA methylation ELISA protocol as described (13, 57).

### Co-IP assay

Cells were harvested and lysed in RIPA buffer (50 mM Tris-HCl (pH 7.5), 150 mM NaCl, 1% NP-40, 5 mM EDTA, and 10% glycerol) containing protease and phosphatase inhibitors. METTL3 complexes were precipitated with METTL3 antibody and Dynabeads protein G (1004D, Invitrogen). The same amount of rabbit IgG was used as the negative control. The beads were washed three times with RIPA buffer and resuspended in LDS sample buffer (NP0007, Invitrogen). Input and IP samples were analyzed by immunoblot.

### m^6^A-SAC-Seq, data deposition, access, and bioinformatics analysis

m^6^A-SAC-seq was performed as previously described (16, 17). Purified mRNA (150 ng) from each sample was used for m^6^A-SAC-seq. The m^6^A-SAC-seq data were deposited in the Gene Expression Omnibus (GEO) with accession number GSE280563 (released on May 1, 2025): https://www.ncbi.nlm.nih.gov/geo/query/acc.cgi?acc=GSE280563.

The “pheatmap,” “ggplot2,” and “ggrepel” R packages were used to identify differentiated m^6^A modifications on cellular genes, with thresholds set at fold change ≥2 with *P* < 0.05. The “ggplot2” R package was employed to visualize GO and KEGG enrichment analyses using Metascape (58), with a threshold of *P* < 0.05 indicating significant enrichment.

### RT-PCR and quantitative PCR (qPCR)

Total RNA was extracted using TRIzol (Invitrogen) or RNeasy Plus Kit (74134, Qiagen). cDNA was synthesized from the extracted RNA using iScript cDNA Synthesis Kit (1708891, Bio-Rad), and qPCR was performed using iTaq Universal SYBR Green Supermix (1725120, Bio-Rad). Primers (Integrated DNA Technologies) sequences are listed in Table S3.

### meRIP and RT-qPCR

Total cellular RNA was isolated using TRIzol (Invitrogen), and its concentration was determined by Nanodrop. Total RNA was resuspended with IP buffer (50 mM Tris-HCl (pH 7.5), 150 mM NaCl, 0.1% NP-40, and RNase Inhibitor). m^6^A antibody (202003, Synaptic Systems) or rabbit IgG (cat, vendor) was used for RNA-IP. The Monarch RNA Cleanup Kit (T2030S, New England Biolabs) was used to purify the enriched RNA. RT-qPCR was conducted to detect target gene enrichment levels. Data analysis was performed using the ΔΔCt method.

### Plasmids

pLentiCRISPR v2 was from Dr. Feng Zhang (Addgene plasmid #52961). pLentiCRISPR v2 sgPLIN3 was constructed by ligating an oligonucleotide duplex (Integrated DNA Technologies) into the BsmBI-v2 site (R0739S, New England Biolabs). pcDNA3 Flag PLIN3 was cloned by synthesizing PLIN3 cDNA sequence (GenScript) into the *Nhe* I and *Xho* I sites of pcDNA3 (Invitrogen). PLIN3 1219C > T mutant was generated from pcDNA3 Flag PLIN3 using the QuikChange II Site-Directed Mutagenesis Kit (200523, Agilent), following the manufacturer’s instructions. Oligonucleotide sequences used were listed in Table S3. Plasmids were confirmed by Sanger Sequencing.

### Generation of PLIN3 KO stable Jurkat cell lines and PLIN3 knockdown primary cells

Jurkat cells were transduced with lentiviruses in the presence of polybrene (10 µg/mL) by spinoculation at 1,200 × *g* for 2 h at 25°C. Transduced cells were cultured in complete RPMI-1640 for 48 h prior to selection with puromycin (1.5 µg/mL). After 7 days of selection, single-cell clones were obtained by limiting dilution. PLIN3 KO Jurkat cells were confirmed by immunoblotting and Sanger sequencing of genomic DNA. Primary CD4^+^ T cells were transduced with lentiviruses in the presence of polybrene (10 µg/mL) by spinoculation at 1,200 × *g* for 2 h at 25°C. The transduced cells were then cultured in complete RPMI-1640 for 24 h before undergoing a second round of transduction. After 48 h of transduction, the efficiency of PLIN3 knockdown was confirmed by immunoblotting. CRISPR-Cas9 ribonucleoprotein (crRNP) electroporation in primary activated CD4^+^ T cells was performed as previously described (59). Synthetic crRNA, trans-activating crRNA (tracrRNA), and recombinant Cas9 protein were purchased from Integrated DNA Technologies. Briefly, activated primary CD4^+^ T cells were resuspended in P3 buffer (Lonza) at a density of 5 × 10^5^ cells per reaction and mixed with the crRNP complex. Electroporation was performed using the 4D-Nucleofector System (Lonza) with program EH115. After electroporation, cells were immediately transferred to pre-warmed RPMI-1640 medium containing 20% FBS and incubated at 37°C. Knockout efficiency was evaluated 72 h post-electroporation by immunoblotting. The sequences of control and PLIN3 sgRNA, as well as crRNA, are listed in Table S3.

### HIV-1 infectivity measurement in TZM-bl cell lines

TZM-bl cells (1 × 10^5^) were seeded in 24-well plates overnight and infected with 2 ng p24 HIV-1 stocks. After 48 h, the luciferase activity was measured using ONE-Glo EX Luciferase Assay System (E8120, Promega). Luminescence was quantified using a microplate reader and normalized to total protein content.

### mRNA stability assay

Primary CD4^+^ T cells were mock-treated or infected with HIV-1 at an MOI of 1 for 90 or 96 h. Cells were treated with actinomycin D (10 µg/mL), and total RNA was extracted at the indicated time points. HEK293T cells were transfected with WT or 1219C > T PLIN3 plasmid for 48 h. Cells were treated with actinomycin D (10 µg/mL), and total RNA was extracted at the indicated time points. RT-qPCR was performed to quantify the remaining *PLIN3* mRNA levels. Data analysis was performed using the ΔCt method.

### Nuclear and cytoplasmic RNA fractionation

Nuclear and cytoplasmic RNA fractions of cells were isolated using the Ambion PARIS kit (AM 1921, Thermo Scientific), following the manufacturer’s instructions. The levels of *PLIN3* mRNA, *MALAT1* lncRNA (nuclear RNA marker), and *HPRT* mRNA (cytoplasmic RNA marker) from each fraction were measured by RT-qPCR.

### Polysome fractionation assay

Polysome fractionation was performed as previously described (36). Briefly, 20 million mock and HIV-1-infected primary CD4^+^ T cells were treated with 100 μg/mL cycloheximide (CHX, S7418, Selleckchem) at 37°C for 10 min, washed twice with ice-cold DPBS, and lysed using 400 µL polysome lysis buffer (20 mM Tris HCl, pH 7.4, 140 mM KCl, 10 mM MgCl_2_, 1% Triton X-100) supplemented with 1 mM DTT, 100 ug/mL CHX, 1× protease inhibitor (P8340, Sigma Aldrich), and 100 U/mL Rnase Out (10777019, Invitrogen). The lysates were incubated on ice for 10 min and centrifuged at 4°C, 13,000 × *g* for 10 min. The clear supernatants were moved to pre-chilled tubes, snap frozen in liquid nitrogen, and stored at −80°C. The samples (380 µL) were loaded on top of a linear 10%–50% (wt/vol) sucrose gradients (20 mM Tris HCl, pH 7.4, 140 mM KCl, 10 mM MgCl_2_, 1% Triton X-100, 1 mM DTT, and 100 μg/mL CHX), and centrifuged at 35 K rpm for 2 h at 4°C in a SW-41Ti rotor (Beckman) using Beckman Optima L-90 Ultracentrifuge. Gradients were continuously fractionated into 1 mL volumes using a Biocomp piston fractionator and recording a continuous A_260 nm_ trace with a TRIAX flow cell (Biocomp). Based on the polysome profile, all samples collected from the sucrose gradient were divided into 10 large fractions; 0.2 ng Luciferase Control RNA (L4561, Promega) was added to 400 µL of each fraction. The RNA was extracted using TRIzol. The levels of HIV-1 *gag*, *HPRT*, and *PLIN3* mRNA were measured by RT-qPCR. For each fraction, the target mRNA abundance was normalized to the spike-in control FFLuc mRNA (ΔΔCt method). The abundance of total signal in each fraction was calculated using Qn = 2^ΔΔC^ and p = 100 × Q_n_/Q_total_ as previously described (60).

### SELECT assay

The SELECT assay was conducted as previously described (38). Briefly, HEK293T cells were transfected with PLIN3 WT and 1219C > T plasmids for 48 h using jetPRIME (Sartorius) according to the manufacturer’s instructions. Total cellular RNA was isolated using TRIzol, and 1 µg total RNA was used for annealing. The nucleotide number of *PLIN3* is based on NCBI Reference Sequence NM_005817 (Human *PLIN3* transcript variant 1, mRNA).

### Statistical analysis

Data were analyzed using *t*-test or analysis of variance (ANOVA) with Prism software, and statistical significance was defined as *P* < 0.05.

## Data Availability

All data and materials are included in the article and supplemental material.

## SUPPLEMENTAL MATERIAL

Supplemental figures jvi.01536-25-s0001.pdf Figures S1 to S8; legends for Tables S1 to S3.

Table S1 jvi.01536-25-s0002.xlsx m^6^A-SAC-seq and RNA-seq data of primary CD4^+^ T cells.

Table S2 jvi.01536-25-s0003.xlsx m^6^A-SAC-seq identifies m^6^A modification sites in HIV-1 RNA in HIV-1-infected primary CD4^+^ T cells.

Table S3 jvi.01536-25-s0004.xlsx Sequences of PCR primers, sgRNA, and crRNA used in this study.

ASM does not own the copyrights to Supplemental Material that may be linked to, or accessed through, an article. The authors have granted ASM a non-exclusive, world-wide license to publish the Supplemental Material files. Please contact the corresponding author directly for reuse.
