# Erratum for Huang et al., “Single-base m^6^A epitranscriptomics reveals novel HIV-1 host interaction targets in primary CD4^+^ T cells”

**DOI:** 10.1128/jvi.00344-26

**Published:** 2026-03-09

**Authors:** Siyu Huang, Yutao Zhao, Stacia Phillips, Julia E. Warrick, Michael G. Kearse, Chuan He, Li Wu

## ERRATUM

Volume 99, no. 11, e01536-25, 2025, https://doi.org/10.1128/jvi.01536-25. Abstract, 1st sentence: “in regulating RNA Ostability” should read “in regulating RNA stability.”

