# Supplementary material for: Single-base m6A epitranscriptomics reveals novel HIV-1 host interaction targets in primary CD4+ T cells
Source: J Virol. 2025 Oct 14;99(11):e01536-25. doi: 10.1128/jvi.01536-25 (PMC12645991; doi:10.1128/jvi.01536-25)
Supplement: Supplemental figures — Figures S1 to S8; legends for Tables S1 to S3. [file jvi.01536-25-s0001.pdf]

Supplemental Information

Huang S. et al. Single-base m^{6}A epitranscriptomics reveals novel HIV-1 host interaction targets in primary CD4$^+$ T cells.

Supplemental Figures S1-S8 with legends and Supplemental Table S1-S3 information

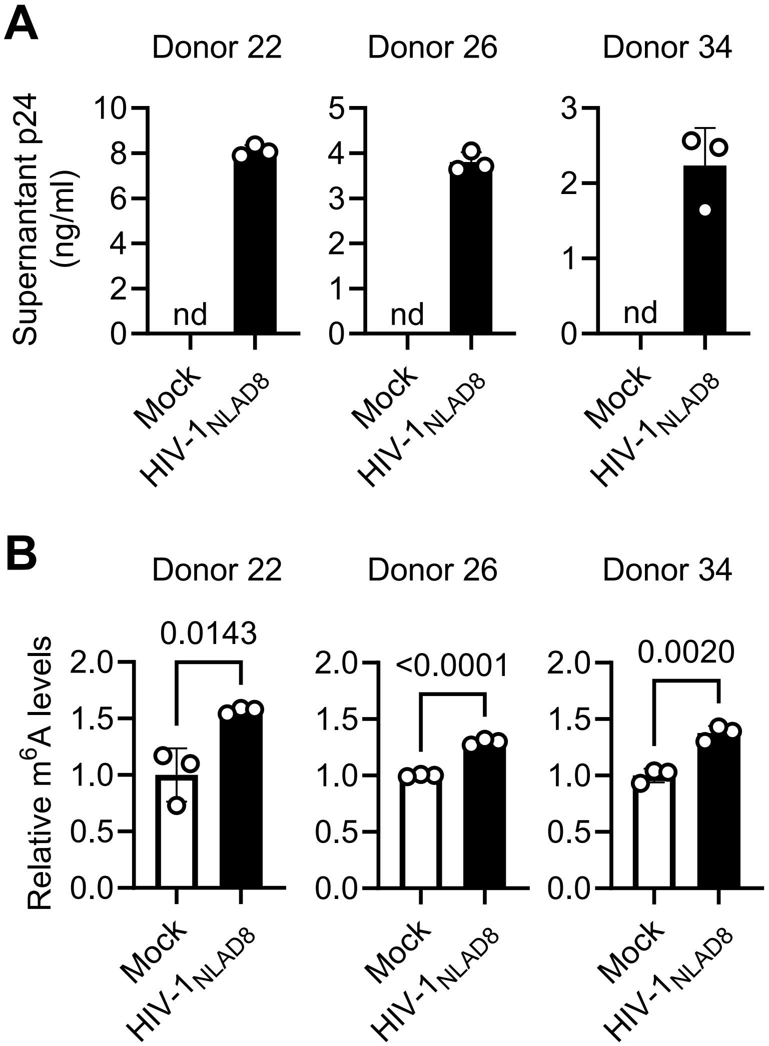

Fig. S1. Infection of R5-tropic HIV-1$_{NLAD8}$ upregulates m^{6}A modification levels in cellular mRNA in primary activated CD4$^+$ T cells. (A-B) Activated primary CD4$^+$ T cells from three heathy donors were mock-infected or infected with HIV-1$_{NLAD8}$ at an MOI of 1 for 96 h. **(A)** HIV-1 infection was confirmed by measuring supernatant p24 levels by ELISA. nd, not detectable. **(B)** m^{6}A levels in cellular mRNA from mock or HIV-1$_{NLAD8}$-infected cells were measured by ELISA. The level of mock control was set as 1. Data are shown as mean ± SD from three individual donors' cells. Two-tailed, unpaired *t*-test (B) were used for statistical analysis (*P* values are shown on figures).

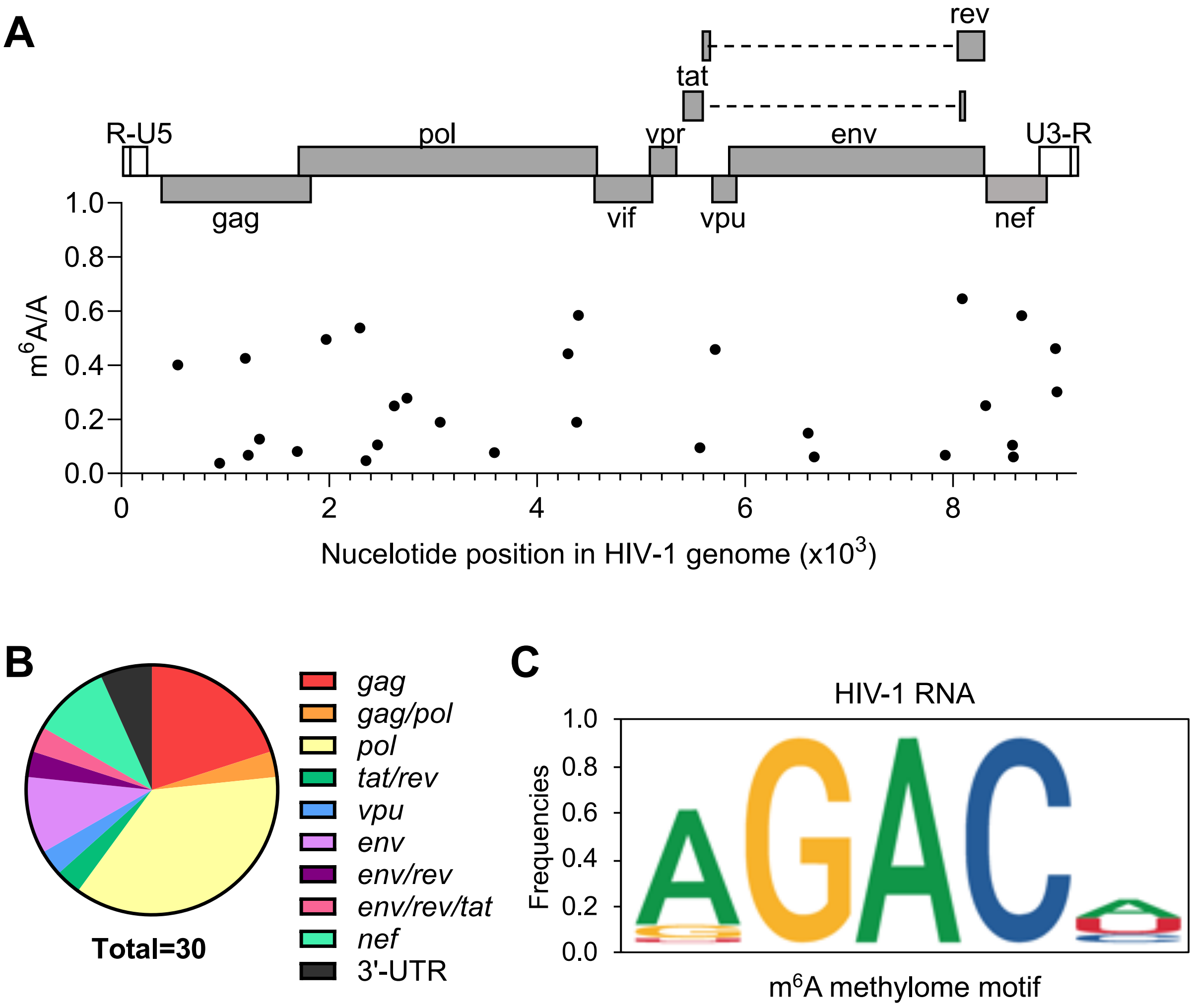

Fig. S2. m^{6}A distribution in HIV-1 genomic RNA. (A) HIV-1 RNA m^{6}A sites and their frequencies are mapped to their nucleotide positions in the HIV-1 genome (GenBank: AF033819.3) **(B)** Distribution of m^{6}A sites in HIV-1 RNA. **(C)** m^{6}A consensus motif frequencies in HIV-1 RNA were determined using m^{6}A-SAC-seq.

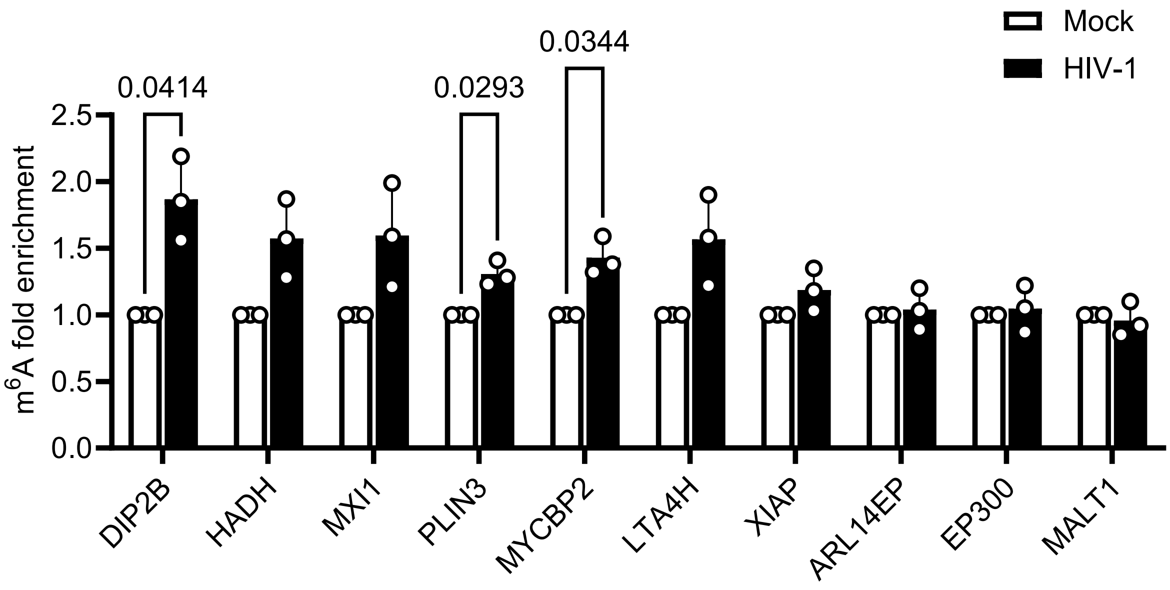

Fig. S3. m^{6}A modification of *PLIN3* mRNA is increased by HIV-1 infection in Jurkat cells. Jurkat cells were mock-infected or infected with HIV-1_{NL4-3} at an MOI of 1 for 72 h. Total cellular RNA was subjected to meRIP, and the enrichment of m^{6}A-modified transcripts in the meRIP was determined relative to mock-infected controls by RT-qPCR. Data are shown as mean ± SD. Multiple unpaired *t*-test was used for statistical analysis (*P* values are shown on figures).

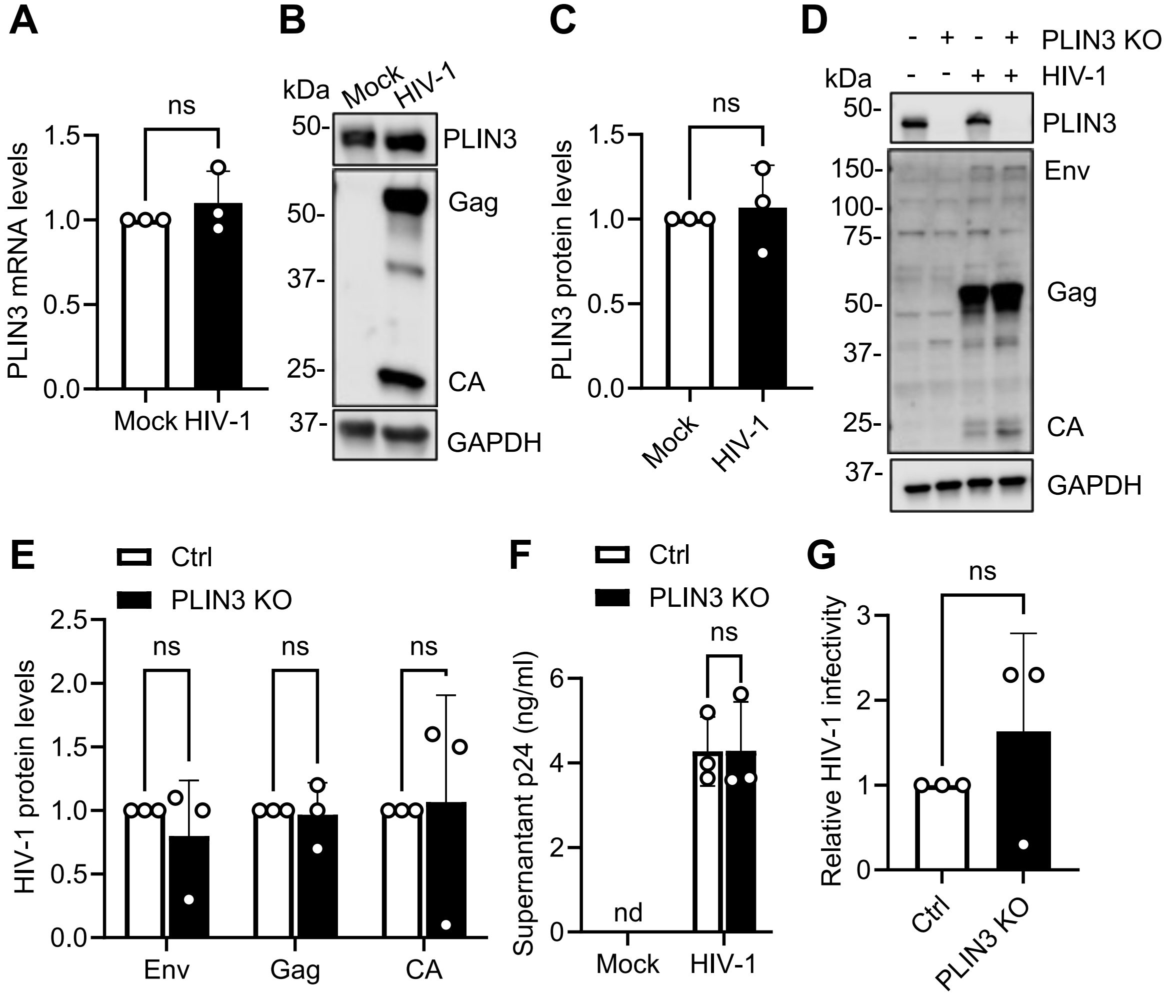

Fig. S4. PLIN3 does not affect HIV-1 replication in Jurkat cells. (A-C) Jurkat cells were mock-infected or infected with HIV-1_{NL4-3} at an MOI of 1 for 72 h. **(A)** *PLIN3* mRNA levels were measured by RT-qPCR. **(B)** PLIN3 and HIV-1 protein expression was measured by IB. A representative IB is shown. **(C)** Relative quantification of PLIN3 protein expression as shown in (B) from three individual experiments. **(D)** Control (Ctrl) and PLIN3 KO Jurkat cells were mock-infected or infected with HIV-1_{NL4-3} at an MOI of 1 for 72 h. PLIN3 and HIV-1 protein expression was measured by IB. One individual experiment result is shown. **(E)** Relative quantification of HIV-1 protein expression as shown in (D) from three independent experiments. **(F)** Cell culture supernatants were collected from Ctrl and PLIN3 KO Jurkat cells with and without HIV-1 infection, and p24 levels were quantified by ELISA. nd, not detectable. **(G)** TZM-bl cells were infected with HIV-1 collected from Ctrl or PLIN3 KO cell culture supernatant. Luciferase activity was measured at 48 hpi. Data are shown as mean ± SD from three individual experiments. Two-tailed, unpaired *t*-test (A, C, F, and G) and multiple unpaired *t*-test (E) were used for statistical analysis. ns, not significant.

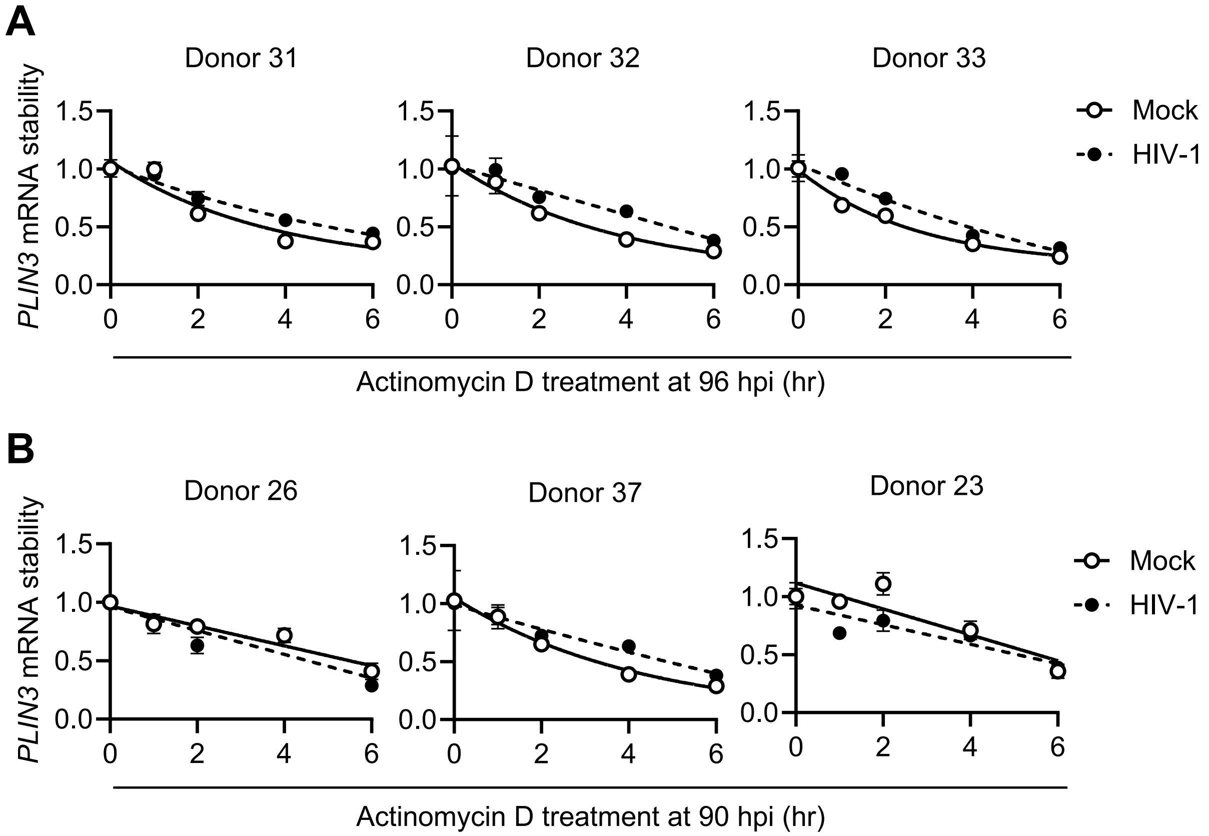

Fig. S5. HIV-1 infection does not affect *PLIN3* mRNA stability in primary CD4+ T cells. Activated primary CD4+ T cells were mock-infected or infected with HIV-1_{NL4-3} at an MOI of 1 for 96 h **(A)** or 90 h **(B)** and then treated with actinomycin D. Samples were collected at the indicated time points, and *PLIN3* mRNA levels were detected by RT-qPCR. Data are shown as mean ± SD of results from three donors' cells. Multiple unpaired *t*-test was used for statistical analysis.

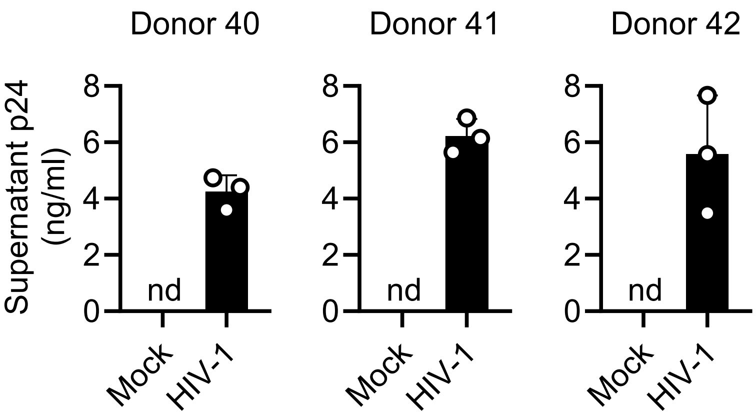

Fig. S6. HIV-1 p24 levels in the supernatants of infected primary CD4⁺ T cells from three donors. Activated primary CD4⁺ T cells from three healthy donors were mock-infected or infected with HIV-$1_{NL4\text{–}3}$ at an MOI of 1 for 96 h before the polysome profile analysis (Fig. 6). HIV-1 replication was confirmed by measuring supernatant p24 levels by ELISA with triplicate samples. nd, not detectable.

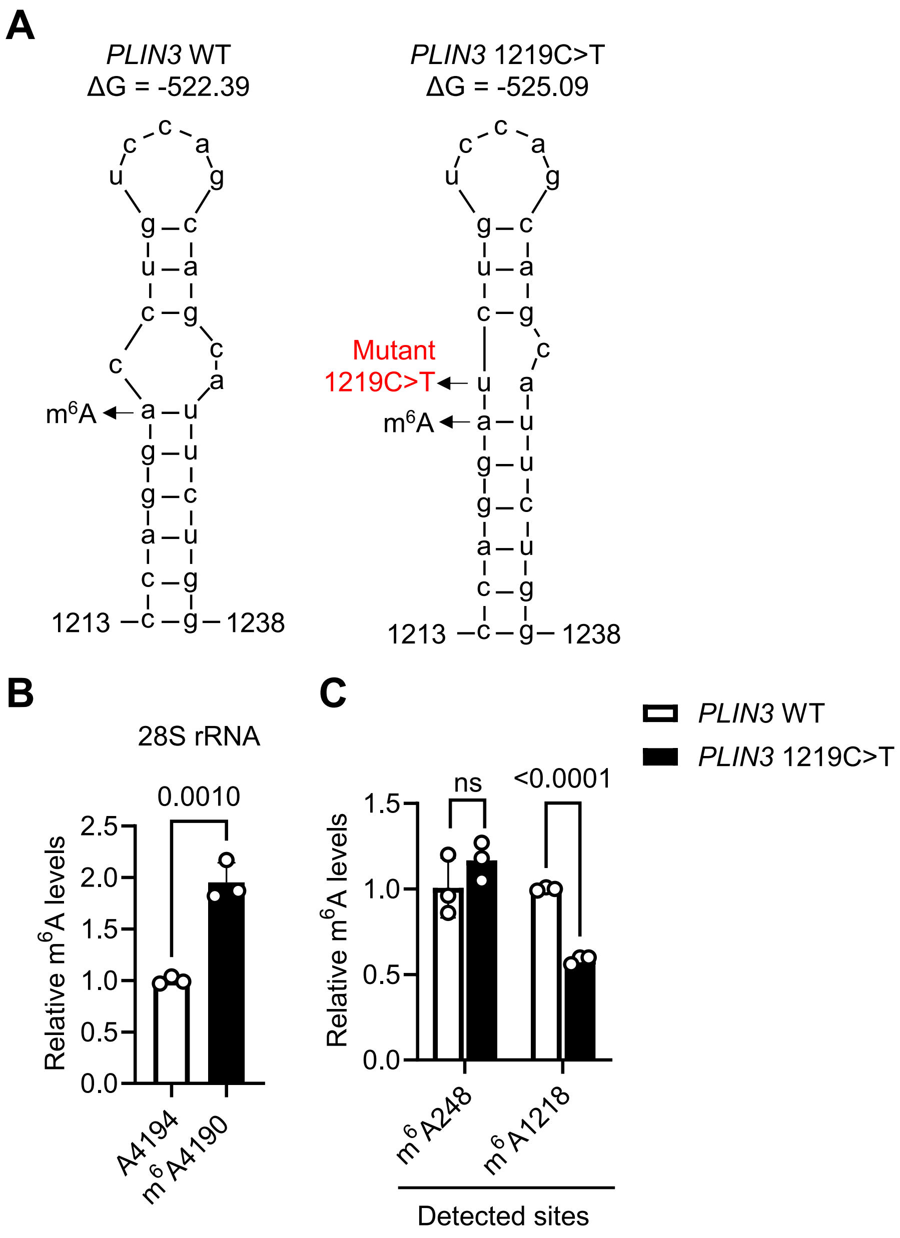

Fig. S7. *PLIN3* 1219C>T mutant shows less m^{6}A modification than PLIN3 WT in HEK293T cells. (A) Predicted secondary structures of the RNA segments of *PLIN3* wild type (WT) and 1219C>T mutant with theoretical ΔG calculated. The m^{6}A1218 site and mutant site are shown with arrows. The nucleotide numbers are based on NCBI Reference Sequence NM_005817 (Human *PLIN3* transcript variant 1, mRNA). **(B and C)** HEK293T cells were transfected with plasmids encoding *PLIN3* WT or 1219C>T mutant for 48 h. Total RNA (1 μg) of HEK293T cells was used for SELECT detection. The bar plots of the relative m^{6}A levels detected by the SELECT assay for two sites (A4194 and m^{6}A4190) of 28S rRNA (B) and two sites (m^{6}A248 and m^{6}A1218) of *PLIN3* RNA (C). The experiments were independently performed twice with similar results, one representative result was shown. Two-tailed unpaired *t*-test (B) or multiple unpaired *t*-test (C) were used for statistical analysis. *P* values are shown on figures. ns, not significant.

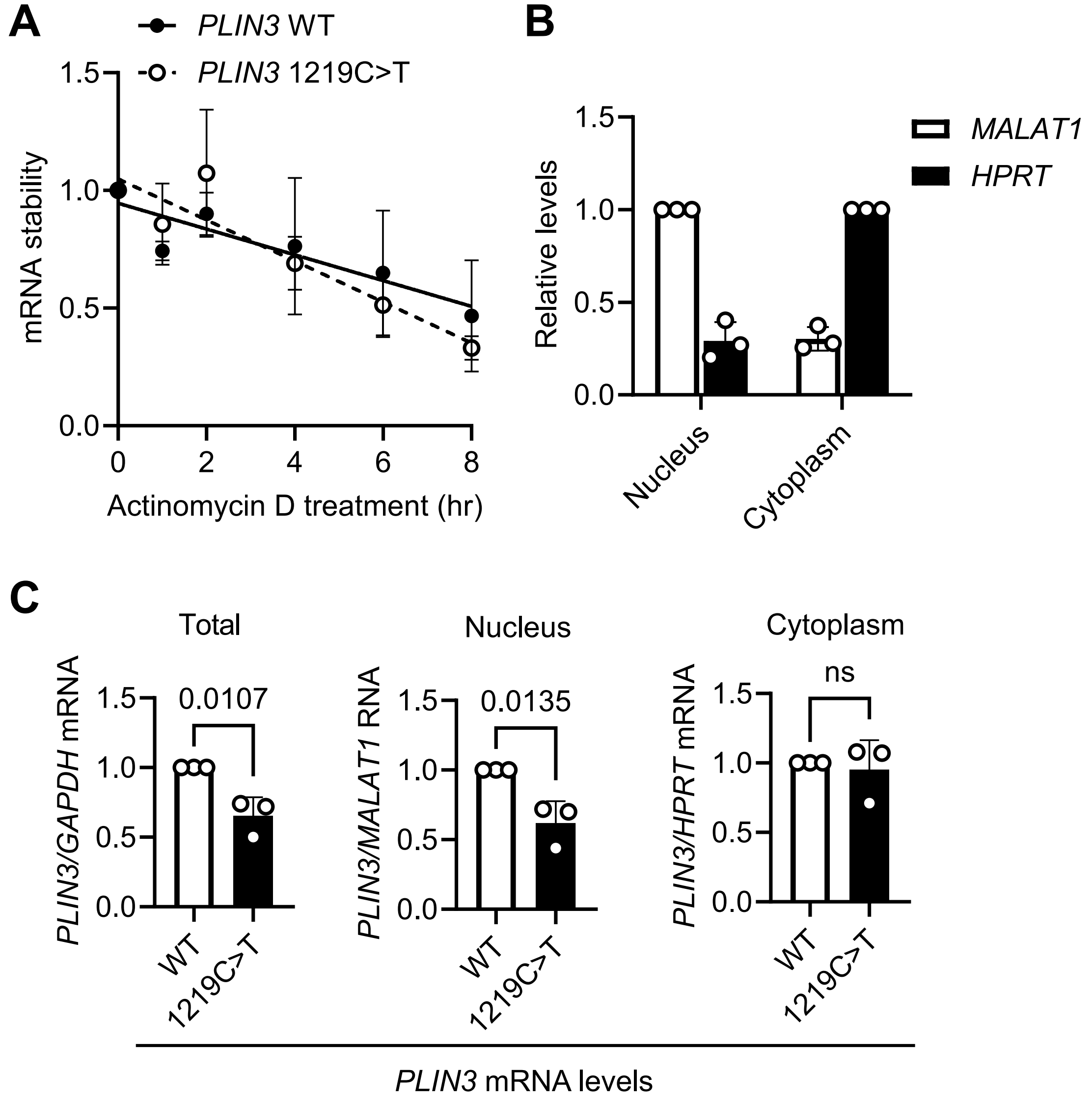

Fig. S8. Mutation of cytosine in the m^{6}A DRACH motif in exon 8 reduces *PLIN3* mRNA expression. (A) HEK293T cells were transfected with plasmids and treated with actinomycin D at 48 h post-transfection. *PLIN3* mRNA levels were measured by RT-qPCR. **(B)** Cellular RNA was separated into nuclear and cytoplasmic fractions prior to RT-qPCR analysis. *MALAT1* lncRNA and *HPRT* mRNA levels from each fraction were measured by RT-qPCR to confirm successful separation of the nucleus and cytoplasm, respectively. **(C)** *PLIN3* mRNA levels in total cell lysates were measured by RT-qPCR and normalized with GAPDH. *PLIN3* mRNA levels from the nuclear and cytoplasmic fractions are shown relative to *MALAT1* and *HPRT*, respectively. Data are shown as means ± SD. Two-tailed unpaired *t*-test was used for statistical analysis. *P* values are shown on the figure. ns, not significant.

Supplemental Table S1-S3 in 3 separate Excel files

Table S1. m^{6}A-SAC-seq and RNA-seq data of primary CD4$^+$ T cells. Cells were infected with Mock or HIV-1 for 96 h and poly(A)-enriched RNA were analyzed based on three individual healthy donors. m^{6}A-SAC-seq and RNA-seq data are in two separate sheets in one Excel file. Data of genes listed in Table 1 are highlighted in red (m^{6}A-SAC-seq).

Table S2. m^{6}A-SAC-seq identifies m^{6}A modification sites in HIV-1 RNA in HIV-1 infected primary CD4$^+$ T cells. Cells were infected with Mock or HIV-1 for 96 h and poly(A)-enriched RNA were analyzed based on three individual healthy donors. m^{6}A motifs, m^{6}A/A ratio of individual samples and their average values are included in the Excel file.

Table S3. Sequences of PCR primers, sgRNA, and crRNA used in this study.
